# Assessing the role of genome-wide DNA methylation between smoking and risk of lung cancer using repeated measurements: the HUNT study

**DOI:** 10.1093/ije/dyab044

**Published:** 2021-03-17

**Authors:** Yi-Qian Sun, Rebecca C Richmond, Matthew Suderman, Josine L Min, Thomas Battram, Arnar Flatberg, Vidar Beisvag, Therese Haugdahl Nøst, Florence Guida, Lin Jiang, Sissel Gyrid Freim Wahl, Arnulf Langhammer, Frank Skorpen, Rosie M Walker, Andrew D Bretherick, Yanni Zeng, Yue Chen, Mattias Johansson, Torkjel M Sandanger, Caroline L Relton, Xiao-Mei Mai

**Affiliations:** 1 Department of Clinical and Molecular Medicine, Norwegian University of Science and Technology, Trondheim, Norway; 2 Department of Pathology, Clinic of Laboratory Medicine, St Olav’s University Hospital, Trondheim, Norway; 3 Center for Oral Health Services and Research Mid-Norway (TkMidt), Trondheim, Norway; 4 MRC Integrative Epidemiology Unit, Population Health Sciences, Bristol Medical School, University of Bristol, Bristol, UK; 5 Central Administration, St Olav’s University Hospital, Trondheim, Norway; 6 Department of Community Medicine, Faculty of Health Sciences, Arctic University of Norway, Tromsø, Norway; 7 K.G. Jebsen Center for Genetic Epidemiology, Department of Public Health and Nursing, Norwegian University of Science and Technology, Trondheim, Norway; 8 Genetic Epidemiology Group, International Agency for Research on Cancer, Lyon, France; 9 Department of Public Health and Nursing, Norwegian University of Science and Technology, Trondheim, Norway; 10 HUNT Research Centre, Department of Public Health and Nursing, Norwegian University of Science and Technology, Trondheim, Norway; 11 Centre for Genomic and Experimental Medicine, Institute of Genetics and Molecular Medicine, University of Edinburgh, Edinburgh, UK; 12 MRC Human Genetics Unit, Institute of Genetics and Molecular Medicine, University of Edinburgh, Western General Hospital, Edinburgh, UK; 13 Faculty of Forensic Medicine, Zhongshan School of Medicine, Sun Yat-Sen University, Guangzhou, China; 14 School of Epidemiology and Public Health, Faculty of Medicine, University of Ottawa, Ottawa, ON, Canada

**Keywords:** Causal inference, EWAS, Mendelian randomization

## Abstract

**Background:**

It is unclear if smoking-related DNA methylation represents a causal pathway between smoking and risk of lung cancer. We sought to identify novel smoking-related DNA methylation sites in blood, with repeated measurements, and to appraise the putative role of DNA methylation in the pathway between smoking and lung cancer development.

**Methods:**

We derived a nested case-control study from the Trøndelag Health Study (HUNT), including 140 incident patients who developed lung cancer during 2009–13 and 140 controls. We profiled 850 K DNA methylation sites (Illumina Infinium EPIC array) in DNA extracted from blood that was collected in HUNT2 (1995–97) and HUNT3 (2006–08) for the same individuals. Epigenome-wide association studies (EWAS) were performed for a detailed smoking phenotype and for lung cancer. Two-step Mendelian randomization (MR) analyses were performed to assess the potential causal effect of smoking on DNA methylation as well as of DNA methylation (13 sites as putative mediators) on risk of lung cancer.

**Results:**

The EWAS for smoking in HUNT2 identified associations at 76 DNA methylation sites (*P *<* *5 × 10^–8^), including 16 novel sites. Smoking was associated with DNA hypomethylation in a dose-response relationship among 83% of the 76 sites, which was confirmed by analyses using repeated measurements from blood that was collected at 11 years apart for the same individuals. Two-step MR analyses showed evidence for a causal effect of smoking on DNA methylation but no evidence for a causal link between DNA methylation and the risk of lung cancer.

**Conclusions:**

DNA methylation modifications in blood did not seem to represent a causal pathway linking smoking and the lung cancer risk.

**Key Messages**

- It was unclear if smoking-related DNA methylation represents a causal pathway for the effect of smoking on the risk of lung cancer.
- This study identified 16 novel smoking-related DNA methylation signals. It provided further evidence that there was no causal effect of DNA methylation in blood on lung cancer risk, by including more and novel DNA methylation sites.
- This is the first study to apply repeated measurements of DNA methylation in blood analysed by MethylationEPIC BeadChip (850K) to identify smoking-related DNA methylation sites.
- It is one of the few studies to assess the causal pathway between smoking, DNA methylation in blood, and the risk of lung cancer.

## Introduction

Lung cancer has been the most common cancer type for several decades worldwide, and it kills the largest number of people with a 5-year survival rate of 10% globally.^1^ Clinical diagnostics are challenging when nodules ≤8 mm are found in the lungs of patient,s as such nodules may not be due to a malignant disease.^2^ Moreover, it is difficult and not without risk to obtain tissue samples from such nodules, and usually these patients are followed up with computed tomography surveillance over time. As a supplement to current standard procedures, it is important to identify biomarkers that are associated with the risk even before cancerous changes arise.^3^ In line with this, recent research has shed light on the involvement of epigenetic modifications in cancer development.^4–6^ Among the epigenetic modifications, DNA methylation involving the addition of a methyl group to the carbon-5 of a cytosine residue, which occurs predominantly at CpG sites (regions of DNA where a cytosine nucleotide is followed by a guanine nucleotide along DNA’s 5’ to 3’ direction) is of particular interest as a molecular mechanism underlying cancer risk.^7^

DNA methylation in blood is highly sensitive to lifestyle influences such as smoking,^8–11^ and emerging evidence suggests that it may also reflect changes in the target tissue such as in the lung.^12^ Recently, Fasanelli *et al.* reported that hypomethylation of smoking-related genes in blood was associated with future onset of lung cancer.^5^ Since tobacco smoking is a causal risk factor of lung cancer,^13^ it is possible that DNA methylation changes lie on the causal pathway between smoke exposure and lung cancer risk. There have been some previous attempts to determine if DNA methylation mediates the influence of lifestyle factors on diseases.^5^^,^^14^ Fasanelli *et al.* suggested that hypomethylation in smoking-related genes *AHRR* and *F2RL3* mediated the effect of tobacco on lung cancer risk with large magnitude.^5^ This study, however, used observational methods that often have limitations such as confounding and reverse causation and thus make causal inference difficult. A Mendelian randomization (MR) approach can be applied in this context, as it has been developed to evaluate causal relationships by using genetic variants as instrumental variables for the exposure of interest.^15^^,^^16^ Genetic variants at a given locus may influence methylation pattern across an extended genomic region.^17^ These variants are defined as methylation quantitative trait loci (mQTLs), and can be used as a proxy for methylation levels in an MR analysis.^18–20^

In this study, we performed epigenome-wide association studies (EWAS) for smoking and lung cancer with repeatedly measured DNA methylation obtained from pre-diagnostic blood samples. The DNA methylation was assayed using the Infinium MethylationEPIC BeadChip (Illumina Inc., CA, USA), which can detect >850 K methylation sites. This supersedes the Illumina Infinium HumanMethylation450 array which has been used in previous EWAS for smoking and lung cancer.^5^^,^^14^^,^^21^^,^^22^ We also performed two-step MR analyses^20^ to appraise the putative causal role of DNA methylation in the pathway between smoking and lung cancer development.

## Methods

All participants gave their informed consent for participation in HUNT. The current study was approved by the Norwegian Regional Committees for Medical and Health Research Ethics (REK 2015/78). Ethical approval for Generation Scotland was obtained from the Tayside Committee on Medical Research Ethics (on behalf of the National Health Service).

### Study design and population

**Figure 1:**
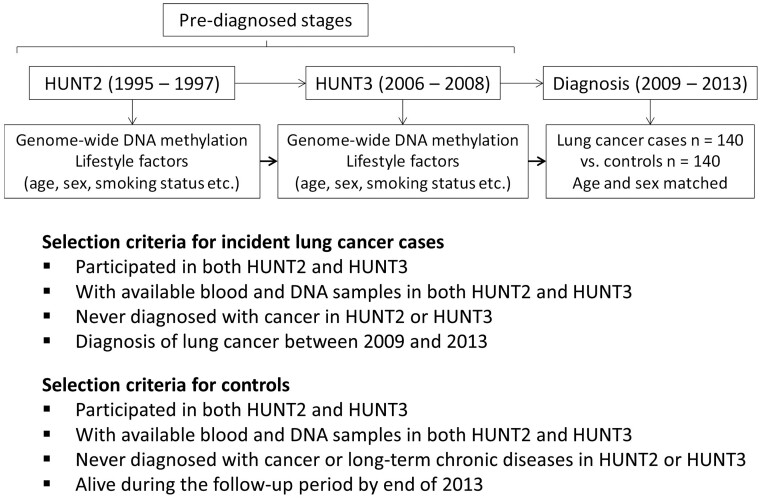
Study design of the nested case-control study from the Trøndelag Health Study (HUNT)

The Trøndelag Health Study (the HUNT Study) is one of the largest population-based health surveys conducted in Norway.^23^ The HUNT Study invited all inhabitants aged 20 years or older in the northern area of Trøndelag in four waves: HUNT1 (1984–86), HUNT2 (1995–97), HUNT3 (2006–08) and HUNT4 (2017–19). A nested case-control study was designed within HUNT2 and HUNT3, including 140 incident cases who developed lung cancer during 2009–13 and 140 age- (±3 years) and sex-matched controls. The study design and selection criteria for cases and controls are described in Figure 1. Incident lung cancer cases were ascertained based on the linkage of data between HUNT and the Cancer Registry of Norway. Pre-diagnostic blood samples were collected in HUNT2 and HUNT3 from both the cases and the controls and stored at −80°C for later use. Among the incident cases, the mean years from blood collection to lung cancer diagnosis were 15.0 (range: 11.8–18.0) in HUNT2 and 3.8 (range: 1.0–6.7) in HUNT3.

### Genotype and lifestyle variables

Information on genotypes and lifestyle factors was extracted from the HUNT databank.^24^ Information on smoking was collected in both HUNT2 and HUNT3. A smoking phenotype (seven levels) was generated taking into account the smoking status and pack-years (pyrs): 0: never smokers; 1: former ≤10.0 pyrs; 2: former 10.1–20.0 pyrs; 3: former ≥20.1 pyrs; 4: current ≤10.0 pyrs; 5: current 10.1–20.0 pyrs; and 6: current ≥20.1 pyrs. A variable for change in smoking status between HUNT2 and HUNT3 [0: decrease (current to former smokers); 1: no change (never to never and former to former); and 2: increase (never to former, never to current, former to current and current to current)] was generated based on status of never, former and current smokers in the HUNT2 and HUNT3. Current to current was classified as an increase in smoking status as exposure to tobacco smoke had been accumulated.

### Genome-wide DNA methylation analysis, quality control and normalization

Genome-wide DNA methylation was analysed in a total of 560 pre-diagnostic blood samples that were collected from 280 study subjects on two occasions when they participated in HUNT2 and HUNT3. About 500 ng DNA isolated from peripheral blood cells was subject to bisulphite conversion, using the EZ DNA methylation kit (Zymo Research, CA, USA). Further, the DNA methylation state of over 850 K DNA methylation sites was quantified using the Infinium MethylationEPIC BeadChip kit (Illumina Inc., CA, USA), according to manufacturer’s instructions. The Bead Chip was imaged on a HiScan System (Illumina, CA, USA) and intensity values (IDAT files) were extracted. The quality control (QC) and functional normalization of the DNA methylation data are described in detail in Supplementary Material and Supplementary Figure S1, available as Supplementary data at *IJE* online. After QC and functional normalization, 864 674 DNA methylation sites in 542 samples (139 cases and 137 controls in HUNT2, 131 cases and 135 controls in HUNT3) remained for the downstream analyses. Normalized DNA methylation estimates were presented as beta-values, ranging from 0 to 1.

### Statistical analysis

All statistical analyses were performed with R (version 3.6.1) or Stata/SE 15.1 (StataCorp, College Station, TX). A detailed description of the statistical analyses is given in the online Supplementary Material. Different sets of data that were used for specific statistical analyses are described in Supplementary Table S1, available as Supplementary data at *IJE* online.

First, we carried out an EWAS for the smoking phenotype (the seven levels) in blood samples collected from the controls in HUNT2. Linear regressions were performed with DNA methylation beta-values as the outcome and smoking phenotype as the exposure. Covariates were included in the linear regression models to adjust for the effects of sex, age and estimated cell counts. Surrogate variable analysis (SVA)^25^ was used to generate 12 variables that were also included as covariates in the EWAS models to adjust for batch and other technical artefacts. The *P*-value cut-off was set at epigenome-wide level (5 × 10^–8^). EWAS for smoking, performed with R package meffil (version 1.1.0).^26^

Second, to confirm the associations identified from the EWAS for smoking, we performed an analysis using repeatedly measured DNA methylation data from both the HUNT2 and the HUNT3 samples (about 11 years apart) in relation to the smoking phenotype in HUNT2 among the controls. A less computationally intensive strategy with cluster-robust standard errors (LMRSE) was performed.^27^ We also explored the possible effect of change in smoking status between HUNT2 and HUNT3 (categorized as decrease, no change or increase) on change in DNA methylation (beta-value of DNA methylation in HUNT3 minus beta-value of DNA methylation in HUNT2) among the controls.

Third, EWAS for lung cancer was performed among the lung cancer cases vs controls with DNA methylation as the exposure measured in HUNT2 and HUNT3, respectively, and the *P*-value cut-off was set at 5 × 10^–8^.

Fourth, the smoking-related DNA methylation sites that overlapped between the EWAS for smoking and the EWAS for lung cancer in the HUNT2 samples were individually evaluated as potential mediators between the smoking phenotype and lung cancer, using mediation analysis. Multiple mediators were then considered simultaneously, and a weighted methylation score was calculated.

Fifth, two-step MR analyses were performed. A first step was applied to evaluate the causal effect of smoking on DNA methylation. We used a smoking genetic score including three single nucleotide polymorphisms (SNPs) as an instrumental variable for the smoking phenotype: rs6265 (*BDNF*) associated with smoking initiation, rs1051730 (*CHRNA3*) with smoking quantity and rs3025343 (*DBH*) with smoking cessation.^28^ One-sample MR using the two-stage least square (2SLS) method was applied to investigate a causal relationship between smoking and DNA methylation at the sites identified in the EWAS for smoking. A second-step MR was performed to evaluate the putative causal association between DNA methylation and the risk of lung cancer. We applied a two-sample MR in order to leverage power from large genome-wide association studies (GWAS). Instruments for the DNA methylation sites detected as putative mediators with the mediation analyses were extracted from an mQTL (both *cis* and *trans*) GWAS in a subset of Generation Scotland (*n* = 5101).^19^^,^^29^ Summary statistics of lung cancer GWAS were derived from McKay *et al*.^30^ with sample size 85 716 (cases 29 266 vs controls 56 450). The inverse-variance weighted (IVW) method or Wald ratio method (when only one mQTL as instrumental variable) was used to calculate the causal estimates.

## Results

### Characteristics of study participants

Characteristics of the lung cancer cases and controls whose DNA methylation was measured in HUNT2 and HUNT3 and passed QC are presented in Supplementary Table S2, available as Supplementary data at *IJE* online. There were more men than women (55% vs 45%). The mean age was similar in cases and controls (56.4 vs 55.6 years in HUNT2 and 67.8 vs 66.8 years in HUNT3). About 90% of the lung cancer cases were former or current smokers whereas about half of the controls were never smokers in HUNT2 and HUNT3.

### Identification of DNA methylation sites associated with smoking

**Figure 2:**
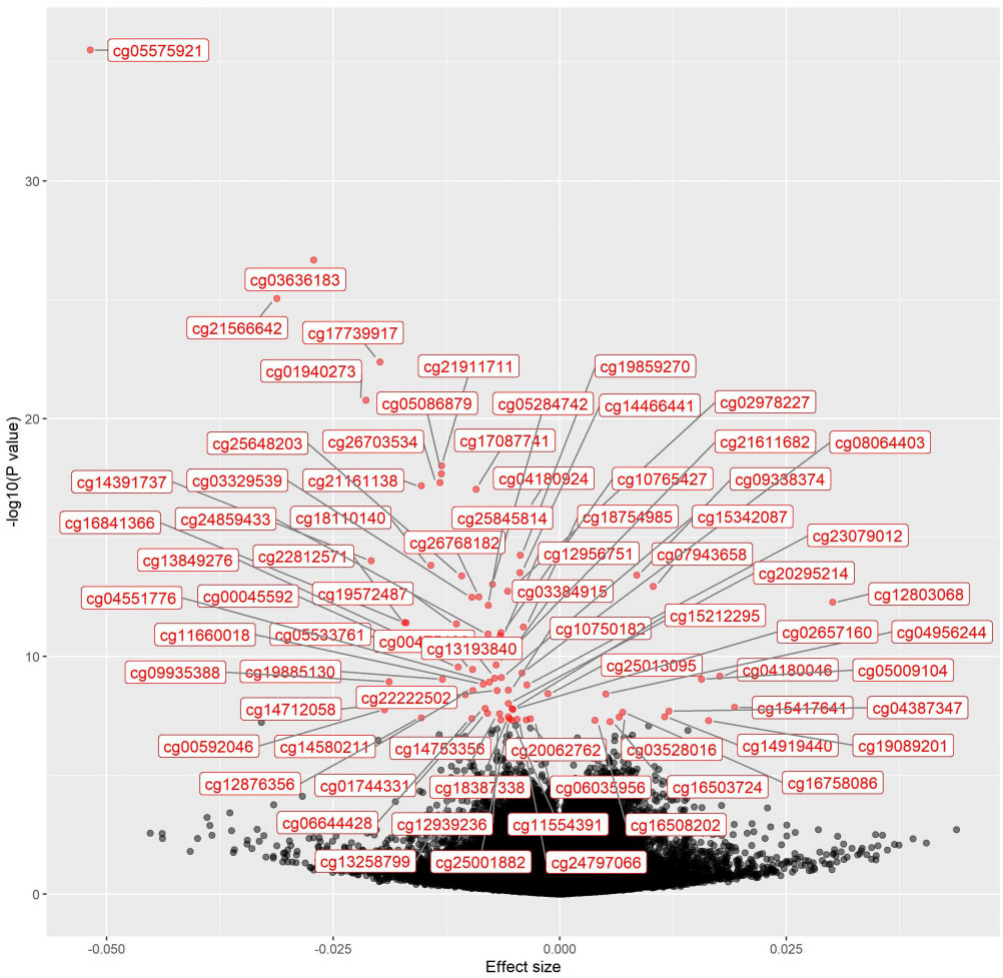
Associations between smoking and genome-wide DNA methylation in blood samples collected in HUNT2 in controls (*n* = 128). Red dots with labels of DNA methylation sites: *P *<* *5 × 10^–8^. Effect size stands for beta value of DNA methylation per level increase of the smoking phenotype (seven levels). HUNT: the Trøndelag Health Study

**Figure 3:**
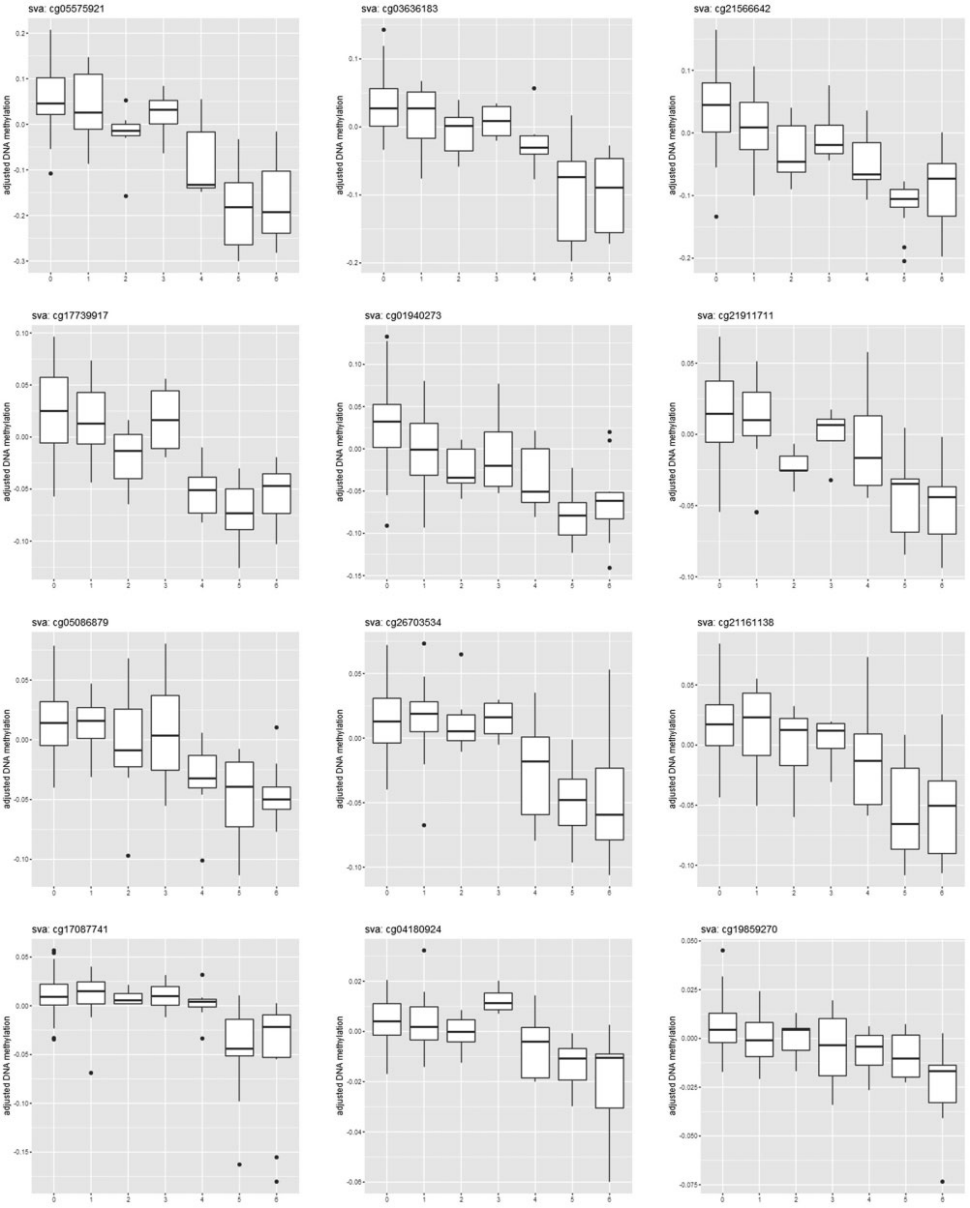
Associations between smoking (horizontal axis) and DNA methylation (vertical axis) for the top 12 smoking-related DNA methylation sites. X-axis represents seven levels of smoking phenotype [0: never smokers; 1: former ≤10.0 pack-years (pyrs); 2: former 10.1–20.0 pyrs; 3: former ≥20.1 pyrs; 4: current ≤10.0 pyrs; 5: current 10.1–20.0 pyrs; 6: current ≥20.1 pyrs]. sva: surrogate variable analysis

The EWAS for smoking in blood samples collected in HUNT2 was performed in 128 of the 137 controls, due to missing data on the smoking phenotype. We identified 76 (*P *<* *5 × 10^–8^) DNA methylation sites (Table 1 and Figure 2; Supplementary Figure S2, available as Supplementary data at *IJE* online). The range of the effect sizes (difference in DNA methylation beta-value per one level increase in smoking phenotype) was from -0.052 to 0.030. Smoking was inversely associated with DNA methylation for 63 (83%) of the 76 sites, among which cg05575921 had the strongest association (*P *=* *3.0 × 10^–36^). Top DNA methylation sites around or within genes (5’-UTR or gene body) such as *AHRR*, *F2RL3*, *RARA*, *MGAT3*, *GPR15* and *PRSS23*, were identified as being associated with smoking. Box plots showed a dose-response association between the smoking phenotype and DNA hypomethylation for most of the 12 top sites (*P-*values <5.5 × 10^–15^, Figure 3).

**Table 1:** Epigenome-wide associations (*P *<* *5 × 10^–8^) for smoking in blood samples collected in HUNT2 in controls (**n** = 128)

DNAm sites	Coefficient^a^	*P*-value	Chr	Position^b^	Gene	Gene region	Exclusively in EPIC Beadchip	Novel DNAm sites and loci^c^
cg05575921	−0.052	2.97E-36	5	373378	AHRR	Body		
cg03636183	−0.027	2.07E-27	19	17000585	F2RL3	Body		
cg21566642	−0.031	8.57E-26	2	233284661				
cg17739917	−0.020	4.04E-23	17	38477572	RARA	5’UTR	Yes	
cg01940273	−0.021	1.65E-21	2	233284934				
cg21911711	−0.013	9.32E-19	19	16998668	F2RL3	TSS1500	Yes	
cg05086879	−0.013	2.06E-18	22	39861490	MGAT3	5’UTR	Yes	
cg26703534	−0.013	4.74E-18	5	377358	AHRR	Body		
cg21161138	−0.015	6.53E-18	5	399360	AHRR	Body		
cg17087741	−0.009	8.98E-18	2	233283010				
cg04180924	−0.003	6.53E-17	3	98272064			Yes	Yes
cg19859270	−0.004	5.35E-15	3	98251294	GPR15	1^st^ Exon		
cg14391737	−0.021	9.17E-15	11	86513429	PRSS23	5’UTR; Body	Yes	
cg18110140	−0.014	1.47E-14	15	75350380			Yes	Yes
cg14466441	−0.004	2.92E-14	6	11392193			Yes	Yes
cg09338374	0.008	3.73E-14	22	39888390			Yes	Yes
cg25648203	−0.011	3.95E-14	5	395444	AHRR	Body		
cg05284742	−0.007	9.00E-14	14	93552128	ITPK1	Body		
cg07943658	0.010	1.10E-13	5	352001	AHRR	Body	Yes	
cg02978227	−0.006	1.79E-13	3	98292027			Yes	Yes
cg26768182	−0.009	3.10E-13	9	134272679			Yes	Yes
cg03329539	−0.010	3.21E-13	2	233283329				
cg12803068	0.030	5.12E-13	7	45002919	MYO1G	Body		
cg25845814	−0.008	7.08E-13	14	74224613	MIR4505; ELMSAN1	TSS1500; 5’UTR	Yes	
cg16841366	−0.017	3.73E-12	2	233286192			Yes	Yes
cg22812571	−0.017	3.86E-12	2	233286229			Yes	Yes
cg19572487	−0.011	4.20E-12	17	38476024	RARA	5’UTR		
cg18754985	−0.004	5.75E-12	3	98237750	CLDND1	Body		
cg10765427	−0.007	9.99E-12	19	17005225	CPAMD8	Body	Yes	
cg24859433	−0.008	1.13E-11	6	30720203				
cg12956751	−0.007	1.32E-11	2	233246922	ALPP	3’UTR	Yes	
cg03384915	−0.005	3.62E-11	19	16986822	SIN3B	Body		
cg05533761	−0.018	4.66E-11	11	86437953			Yes	Yes
cg13849276	−0.013	8.63E-11	17	41328544	NBR1	Body	Yes	Yes
cg21611682	−0.007	2.21E-10	11	68138269	LRP5	Body		
cg00045592	−0.011	2.75E-10	1	160714299	SLAMF7	5’UTR; Body	Yes	Yes
cg00475490	−0.010	3.48E-10	11	86517110	PRSS23	5’UTR; Body	Yes	
cg08064403	−0.004	4.82E-10	3	98240258	CLDND1	Body	Yes	
cg04180046	0.018	6.60E-10	7	45002736	MYO1G	Body		
cg15342087	−0.006	7.68E-10	6	30720209				
cg13193840	−0.007	8.11E-10	2	233285289				
cg05009104	0.016	8.97E-10	7	45002980	MYO1G	Body	Yes	
cg19885130	−0.013	9.05E-10	11	68146832	LRP5	5’UTR; Body	Yes	
cg09935388	−0.019	1.15E-09	1	92947588	GFI1	Body		
cg04551776	−0.008	1.17E-09	5	393366	AHRR	Body		
cg11660018	−0.008	1.43E-09	11	86510915	PRSS23	TSS1500		
cg23079012	−0.004	1.55E-09	2	8343710				
cg10750182	−0.006	2.62E-09	10	73497514	C10orf105; CDH23	5'UTR; 1^st^ Exon; Body		
cg14712058	−0.007	2.63E-09	19	16988083	SIN3B	Body		
cg22222502	−0.010	2.66E-09	5	150161551	SMIM3	5’UTR	Yes	
cg25013095	−0.001	3.63E-09	2	231809672				
cg04956244	0.005	3.74E-09	17	38511592	RARA	Body		
cg14580211	−0.010	4.07E-09	5	150161299	C5orf62	Body		
cg20295214	−0.006	9.28E-09	1	206226794	AVPR1B	Body		
cg15417641	0.019	1.36E-08	3	53700141	CACNA1D	Body		
cg01744331	−0.008	1.53E-08	11	2722358	KCNQ1OT1; KCNQ1	TSS1500; Body		
cg15212295	−0.005	1.55E-08	17	64710687	PRKCA	Body		
cg02657160	−0.005	1.66E-08	3	98311063	CPOX	Body		
cg00592046	−0.019	1.74E-08	18	69848574			Yes	Yes
cg04387347	0.012	1.98E-08	16	88537187	ZFPM1	Body		
cg16758086	0.007	2.17E-08	1	6173356	CHD5	Body	Yes	
cg14753356	−0.008	2.45E-08	6	30720108				
cg13258799	−0.007	2.54E-08	15	28413705	HERC2	Body	Yes	Yes
cg14919440	0.012	3.41E-08	11	113234367	TTC12	Body	Yes	
cg18387338	−0.006	3.45E-08	7	26591438			Yes	Yes
cg03528016	0.007	3.51E-08	2	73871942	ALMS1P	TSS200		
cg12876356	−0.015	3.73E-08	1	92946825	GFI1	Body		
cg06644428	−0.010	4.09E-08	2	233284112				
cg25001882	−0.006	4.20E-08	14	78619077			Yes	Yes
cg06035956	−0.003	4.24E-08	5	379099	AHRR	Body	Yes	
cg24797066	−0.005	4.34E-08	20	48407084			Yes	Yes
cg20062762	−0.004	4.58E-08	14	74207053	ELMSAN1	5’UTR	Yes	
cg12939236	−0.006	4.59E-08	15	40395476	BMF	Body	Yes	
cg16508202	0.004	4.81E-08	7	147501016	CNTNAP2	Body	Yes	
cg11554391	−0.005	4.82E-08	5	321320	AHRR	Body		
cg19089201	0.016	4.91E-08	7	45002287	MYO1G	3’UTR		

3’ UTR, 3’ untranslated region; 5’ UTR, 5’ untranslated region; Chr, chromosome; DNAm, DNA methylation; TSS200, up to 200 nucleotides upstream of transcription start site; TSS1500, 200 to 1500 nucleotides upstream of transcription start site.

a Coefficient: difference in DNA methylation beta-value per level increase in smoking phenotype.

b Based on human genome reference build b37.

c By searching the EWAS catalogue [http://www.ewascatalog.org/] and on the Pubmed per 2020–05-15.

Among the identified 76 sites, 35 sites were exclusive to the MethylationEPIC BeadChip compared with the HumanMethylation450 BeadChip (Table 1). Nineteen of the 35 EPIC BeadChip specific sites confirmed previous smoking loci, such as *F2RL3*, *AHRR*, *MGAT3*, *GPR15*, *PRSS23*, *ELMSAN1* and *RARA* etc. Sixteen DNA methylation sites are novel signals (Table 1), and three of them were annotated to the following genes: *NBR1* (cg13849276, *P *=* *8.7 × 10^–11^), *SLAMF7* (cg00045592, *P *=* *2.8 × 10^–10^) and *HERC2* (cg13258799, *P *=* *2.5 × 10^–8^). The remaining 13 signals were not annotated.

### Confirmation of EWAS for smoking

Among the 76 sites, 75 sites (i.e. except cg23079012) were confirmed after Bonferroni correction (actual *P*-value × 76 < 0.05) using repeated measurements with LMRSE (*n *= 124). The results of LMRSE highly correlated with those of a computationally intensive linear mixed effects model (LMEM) with random intercept for randomly selected 1000 DNA methylation sites (Supplementary Figure S3, available as Supplementary data at *IJE* online: correlation *R *=* *0.97, *P *<* *2.2 × 10^–16^). Estimates from the EWAS for smoking and the LMRSE analysis showed a strong correlation for the 76 sites (*R *=* *0.99, *P *<* *2.2 × 10^–16^, Supplementary Figure S4, available as Supplementary data at *IJE* online).

**Figure 4:**
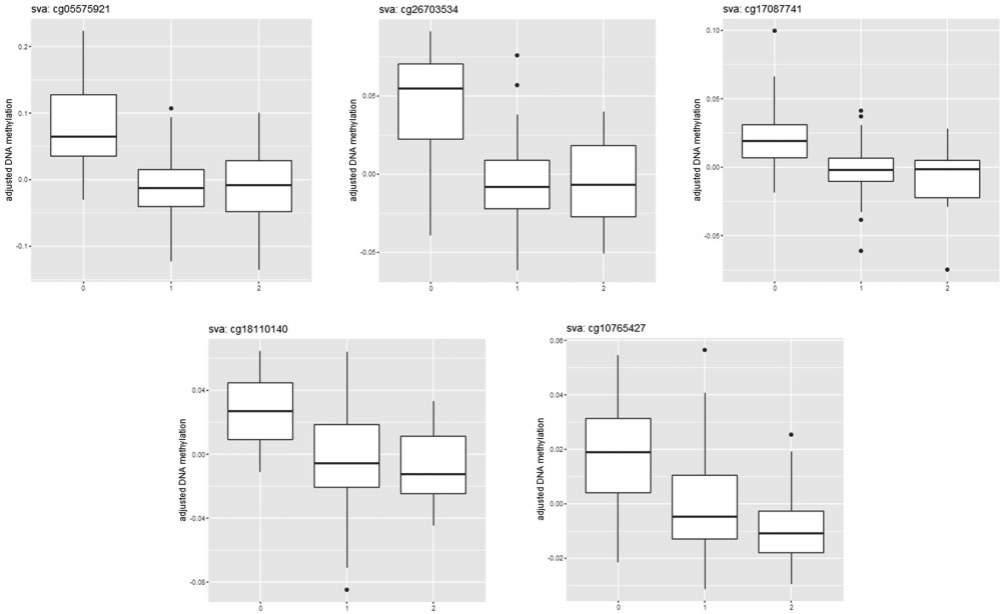
Associations between change in smoking status (horizontal axis) and change in DNA methylation (vertical axis) between HUNT2 and HUNT3 for the five smoking-related DNA methylation sites (Bonferroni corrected *P *<* *0.05). Horizontal axis stands for change in smoking status [0: decrease (from current to former smokers); 1: no change (never to never; former to former); and 2: increase (never to former; never to current; former to current; current to current)]. HUNT: the Trøndelag Health Study; sva: surrogate variable analysis

Change in smoking status between HUNT2 and HUNT3 was available for the 128 controls who were categorized as 16 with decrease (current to former), 88 with no change (59 never to never, 29 former to former), and 24 with increase (3 never to former, 1 never to current, 6 former to current, 14 current to current) in smoking status. Of the 76 DNA methylation sites, five sites were associated with smoking change (Bonferroni corrected *P *<* *0.05) and showed a dose-response relationship (Table 2 and Figure 4). Among the five sites, cg18110140 is a novel site.

**Table 2:** Associations (Bonferroni corrected *P *<* *0.05) between change in smoking status and change in DNA methylation among smoking-related DNA methylation sites^a^

DNAm sites	Coefficient^b^	95% CI		Chromosome	Position	Bonferroni corrected *P*-value
cg05575921	−0.037	−0.053	−0.021	5	373378	1.20E-03
cg26703534	−0.020	−0.029	−0.011	5	377358	1.47E-03
cg17087741	−0.012	−0.017	−0.006	2	233283010	2.80E-03
cg18110140	−0.015	−0.023	−0.007	15	75350380	2.06E-02
cg10765427	−0.012	−0.017	−0.007	19	17005225	8.50E-04

DNAm, DNA methylation.

a Smoking-related DNA methylation sites: the 76 sites were identified in EWAS for smoking in the cross-sectional analysis.

b Coefficient: difference in the change of DNA methylation beta-value per level increase in smoking change.

### Identification of DNA methylation sites associated with lung cancer

When the smoking phenotype was included in the EWAS model for lung cancer, no DNA methylation sites survived adjustment for multiple tests (*P *<* *5 × 10^–8^) in either HUNT2 (139 cases vs 137 controls) or HUNT3 (131 cases vs 135 controls). When smoking was not included in the EWAS model to study DNA methylation sites as potential mediators linking smoking and lung cancer, associations at 50 and 18 DNA methylation sites survived adjustment for multiple tests in HUNT2 (Table 3; Supplementary Figure S5, available as Supplementary data at *IJE* online) and HUNT3, respectively. Of these, 30 sites from HUNT2 and all the 18 sites from HUNT3 overlapped with the 76 smoking-related sites and 17 sites overlapped between HUNT2 and HUNT3.

**Table 3:** Epigenome-wide associations (*P *<* *5 × 10^–8^) for lung cancer in 139 cases vs 137 controls in the HUNT2 study (*n* = 276)

DNAm sites	Coefficient^a^	SE	*P*-value	OR^b^	Chromosome	Position	Gene	Smoking-related DNAm sites
cg05575921	−11.854	1.603	1.43E-13	0.89	5	373378	AHRR	Yes
cg21911711	−33.453	4.815	3.72E-12	0.72	19	16998668	F2RL3	Yes
cg03636183	−18.879	2.728	4.46E-12	0.83	19	17000585	F2RL3	Yes
cg21566642	−17.334	2.512	5.21E-12	0.84	2	233284661		Yes
cg01940273	−23.919	3.474	5.76E-12	0.79	2	233284934		Yes
cg17739917	−24.414	3.601	1.20E-11	0.78	17	38477572	RARA	Yes
cg21161138	−25.790	3.892	3.44E-11	0.77	5	399360	AHRR	Yes
cg24859433	−39.290	5.970	4.66E-11	0.68	6	30720203		Yes
cg19572487	−28.832	4.494	1.40E-10	0.75	17	38476024	RARA	Yes
cg05086879	−27.038	4.216	1.43E-10	0.76	22	39861490	MGAT3	Yes
cg14391737	−18.074	2.837	1.88E-10	0.83	11	86513429	PRSS23	Yes
cg18110140	−24.492	3.849	1.98E-10	0.78	15	75350380		Yes
cg25648203	−28.854	4.557	2.43E-10	0.75	5	395444	AHRR	Yes
cg11931220	−42.253	6.714	3.10E-10	0.66	12	49276387		
cg20174472	−59.947	9.614	4.50E-10	0.55	20	61283288	SLCO4A1	
cg00073090	−54.425	8.762	5.25E-10	0.58	19	1265879		
cg17287155	−41.505	6.754	7.97E-10	0.66	5	393347	AHRR	
cg19859270	−68.254	11.164	9.72E-10	0.51	3	98251294	GPR15	Yes
cg03329539	−30.232	4.971	1.19E-09	0.74	2	233283329		Yes
cg16841366	−17.880	2.947	1.30E-09	0.84	2	233286192		Yes
cg24797066	−46.647	7.695	1.35E-09	0.63	20	48407084		Yes
cg15342087	−34.067	5.665	1.82E-09	0.71	6	30720209		Yes
cg09834951	−51.967	8.660	1.96E-09	0.59	19	1265877		
cg00475490	−34.454	5.825	3.31E-09	0.71	11	86517110	PRSS23	Yes
cg00045592	−25.109	4.250	3.47E-09	0.78	1	160714299	SLAMF7	Yes
cg14466441	−63.619	10.773	3.51E-09	0.53	6	11392193		Yes
cg27537125	−58.260	9.918	4.25E-09	0.56	1	25349681		
cg27241845	−24.766	4.216	4.25E-09	0.78	2	233250370		
cg11660018	−29.072	4.977	5.16E-09	0.75	11	86510915	PRSS23	Yes
cg17668115	−30.842	5.280	5.17E-09	0.73	1	156868625	PEAR1	
cg22812571	−17.320	2.991	7.01E-09	0.84	2	233286229		Yes
cg26271591	−19.184	3.313	7.03E-09	0.83	2	178125956	NFE2L2	
cg25845814	−35.177	6.118	8.96E-09	0.70	14	74224613	MIR4505; ELMSAN1	Yes
cg27650500	−55.569	9.762	1.25E-08	0.57	1	25298480		
cg05284742	−36.017	6.332	1.29E-08	0.70	14	93552128	ITPK1	Yes
cg09935388	−10.988	1.933	1.32E-08	0.90	1	92947588	GFI1	Yes
cg21901790	−38.782	6.840	1.43E-08	0.68	17	46599866		
cg27215690	−37.380	6.594	1.44E-08	0.69	1	25344157		
cg21322436	−32.104	5.671	1.50E-08	0.73	7	145812842	CNTNAP2	
cg04885881	−25.282	4.475	1.61E-08	0.78	1	11123118		
cg00310412	−35.322	6.275	1.82E-08	0.70	15	74724918	SEMA7A	
cg26768182	−29.009	5.162	1.92E-08	0.75	9	134272679		Yes
cg23576855	−6.805	1.216	2.17E-08	0.93	5	373299	AHRR	
cg23771366	−29.786	5.329	2.28E-08	0.74	11	86510998	PRSS23	
cg12939236	−29.866	5.375	2.75E-08	0.74	15	40395476	BMF	Yes
cg25197654	−38.281	6.932	3.34E-08	0.68	8	21914006	DMTN	
cg19885130	−18.171	3.313	4.14E-08	0.83	11	68146832	LRP5	Yes
cg08316204	−45.510	8.303	4.23E-08	0.63	20	35973919	SRC	
cg21611682	−32.264	5.889	4.28E-08	0.72	11	68138269	LRP5	Yes
cg14335029	−37.541	6.865	4.54E-08	0.69	9	134277886		

DNAm, DNA methylation; OR, odds ratio; SE, standard error.

a Coefficient when DNA methylation beta-value changes from 0 to 1; smoking was not adjusted for in the model.

b Odds ratio of lung cancer per 1% increase of DNA methylation at the site.

### Mediation effects of DNA methylation on the pathway between smoking and risk of lung cancer

The 30 smoking- and lung cancer-overlapped DNA methylation sites from HUNT2 were tested as potential mediators between smoking and lung cancer, among which 14 sites were identified. The relative mediation effects of the 14 DNA methylation sites and the weighted mediation score based on the sum of the 14 sites are presented in Table 4. The indirect effect carried by the weighted mediation score accounted for 61% of total effect from smoking phenotype to lung cancer development.

**Table 4:** Mediation effect of 14 DNA methylation sites^a^ between smoking phenotype and risk of lung cancer

DNAm sites	Total effect			Indirect effect					
	Coefficient	95% CI		Coefficient	95% CI		Relative indirect effect^b^	95% CI	
cg19859270	0.74	0.48	0.98	0.19	0.05	0.33	0.26	0.07	0.43
cg05575921	0.71	0.47	0.95	0.36	0.00	0.74	0.51	0.01	1.00
cg25845814^c^	0.72	0.48	0.93	0.15	−0.01	0.35	0.21	−0.01	0.47
cg24859433	0.72	0.47	0.90	0.18	0.06	0.37	0.25	0.08	0.48
cg15342087	0.71	0.48	0.90	0.15	0.00	0.28	0.21	0.01	0.38
cg26768182^c^	0.72	0.49	0.93	0.16	0.00	0.37	0.22	0.00	0.43
cg19572487	0.74	0.51	1.00	0.22	0.11	0.35	0.29	0.13	0.48
cg24797066^c^	0.71	0.49	0.94	0.13	−0.03	0.26	0.18	−0.05	0.37
cg21911711^c^	0.72	0.48	1.00	0.21	0.05	0.42	0.29	0.06	0.63
cg00475490^c^	0.73	0.48	1.02	0.19	0.06	0.37	0.26	0.07	0.50
cg00045592^c^	0.70	0.47	0.96	0.14	0.03	0.31	0.20	0.04	0.40
cg03329539	0.70	0.48	0.93	0.14	0.00	0.31	0.20	0.00	0.41
cg14391737^c^	0.70	0.49	0.93	0.13	−0.04	0.29	0.18	−0.06	0.36
cg21161138	0.69	0.47	0.92	0.16	0.04	0.38	0.23	0.06	0.51
Weighted mediation score^d^	0.74	0.46	1.00	0.45	0.12	0.76	0.61	0.17	0.97

DNAm, DNA methylation.

a 14 DNA methylation sites were identified as mediators individually with the counterfactual framework.

b Indirect effect divided by total effect.

c Exclusively in EPIC Beadchip.

d The sum of methylation beta-value at each of 14 DNA methylation sites weighted by effect size with lung cancer.

### Evaluation of potential causal association between smoking and DNA methylation

**Figure 5:**
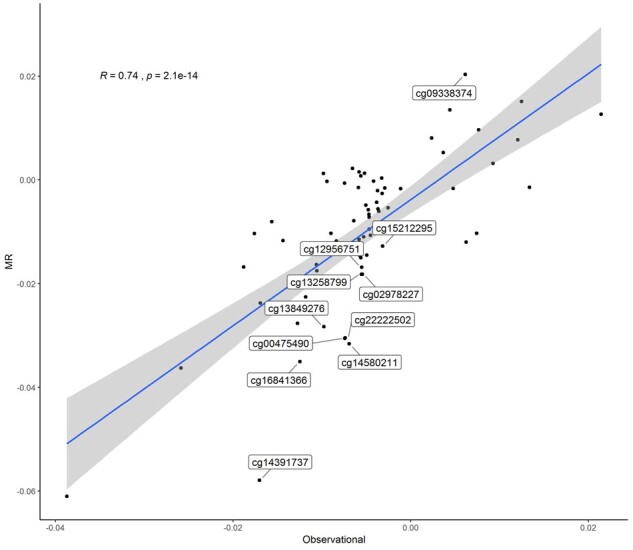
Correlation between Mendelian randomization and epigenome-wide association study estimates for smoking-DNA methylation associations for the smoking-related 76 DNA methylation sites. The 11 sites with *P *<* *0.05 in MR analysis are labelled

Summary statistics from the first-step MR between smoking and DNA methylation for the 76 DNA methylation sites are presented in Supplementary Table S3, available as Supplementary data at *IJE* online. Eleven sites showed statistical evidence for a causal association (*P *<* *0.05). The genetic score explained 1.8% of the variance in smoking with an F statistic of 2.4. To further evaluate the extent to which the EWAS associations reflect causal effects, we plotted the MR estimates against the EWAS estimates for the 76 sites (Figure 5) and it showed a good correlation (*R *=* *0.74, *P *=* *2.1 × 10^–14^).

### Evaluation of putative causal association between DNA methylation and lung cancer risk

The second-step MR evaluated the effect of DNA methylation on risk of lung cancer (Table 5). The 14 putative DNA methylation mediators identified by mediation analysis are linked to genes *GPR15*, *AHRR*, *MIR4505/ELMSAN1*, *RARA*, *F2RL3*, *PRSS23* and *SLAMF7*. We were not able to perform MR for cg24859433 as summary statistics for associations of its mQTLs with lung cancer were not available.^30^ Both *cis* and *trans* mQTLs (range 1 to 9 per DNA methylation site) were used as instrumental variables for DNA methylation. The mQTLs explained 0.6% to 6.8% of the variance in DNA methylation for the included 13 sites. None of the 13 DNA methylation sites demonstrated a causal effect on the risk of lung cancer (Bonferroni correction: actual *P*-value × 13 > 0.05 for all, Table 5). In addition, there was no clear correlation (*R *=* *0.083, *P *=* *0.79) between the estimates derived from the MR and EWAS for lung cancer for the 13 sites (Supplementary Figure S6, available as Supplementary data at *IJE* online). To reduce the possibility of pleiotropy of the instrumental variables, the second-step MR was also performed using *cis*-only mQTLs and it showed no causal evidence (Supplementary Table S4, available as Supplementary data at *IJE* online).

**Table 5:** Second-step Mendelian randomization of DNA methylation and risk of lung cancer

DNAm sites as putative mediators	Chr	Gene	Instrumental variables (mQTLs)	mQTLs chromosome	Variance explained (%)	OR^a^	95% CI		*P*-value
cg19859270^c^	3	GPR15	rs4540316; **rs1529047**; rs1864203; rs6855577; rs6826969; rs74995805; rs150605105	7; 3; 9; 4; 4; 4; 2	2.9	0.93	0.84	1.02	0.13
cg05575921	5	AHRR	**rs11956656**	5	0.9	0.96	0.80	1.16	0.70
cg25845814^b^^,^^c^	14	MIR4505; ELMSAN1	rs1323124; rs111686083; rs139016638; rs951574; rs80186749; rs74959723; **rs112116518**; rs3756764; rs141965025	1; 14; 2; 16; 2; 2; 1; 5; 20	3.7	1.04	0.93	1.16	0.53
cg15342087	6		rs11190127	10	0.7	1.08	0.88	1.32	0.45
cg26768182^b^	9		**rs78581928**	9	0.9	1.23	1.05	1.46	0.01
cg19572487	17	RARA	rs17032705	4	0.8	0.97	0.80	1.18	0.79
cg24797066^b^	20		**rs602598**	20	0.6	1.00	0.80	1.25	0.99
cg21911711^b^	19	F2RL3	**rs56298289**; rs79977579; rs3848656	19; 12; 19	6.8	0.98	0.91	1.06	0.64
cg00475490^b^	11	PRSS23	**rs2279046**	11	3.3	1.05	0.96	1.16	0.25
cg00045592^b^	1	SLAMF7	**rs3766373**; rs352684	1; 1	2.4	0.96	0.86	1.08	0.52
cg03329539	2		**rs13023370**	2	4.0	1.01	0.93	1.10	0.85
cg14391737^b^^,^^c^	11	PRSS23	rs7607726; rs7606236; rs62010937; rs9424468; rs147426883	2; 2; 15; 1; 19	2.1	0.99	0.88	1.12	0.91
cg21161138^c^	5	AHRR	rs62289477; rs117666260; rs79694935; **rs2466287**; rs79991330	3; 8; 4; 5; 10	2.2	1.05	0.95	1.17	0.31

**Bold type:** sentinel mQTL (*cis* mQTL with smallest *P*-value in Generation Scotland).

Chr, chromosome; DNAm, DNA methylation; mQTL, methylation quantitative trait locus.

a Per 1-unit increase of DNA methylation M-value.

b Exclusively in EPIC Beadchip.

c *P *<* *1 × 10^–5^ for association with the mQTLs as no mQTLs found if smaller *P*-value was set.

## Discussion

### Main findings

In this study, we identified 76 DNA methylation sites associated with smoking, using the Illumina Infinium MethylationEPIC BeadChip, among which 16 sites were novel and not captured on the older HumanMethylation450 array. Our results showed that smoking appeared to be a causal factor for DNA methylation modifications in the blood. There was no evidence for a causal effect of smoking-related DNA methylation on the risk of lung cancer.

### Comparison with previous studies

To our knowledge, this is the first study to use the MethylationEPIC BeadChip to identify smoking-related DNA methylation sites in the blood. Of the 76 sites, we replicated 41 sites that were previously identified with 450 K.^8–11^ New probes on 850 K further confirmed some previously identified smoking-related genes. Although it is difficult to compare effect sizes in our study with those in previous work, due to different definitions of smoking phenotype, the genes associated with our top DNA methylation sites are consistent with those frequently found in previous 450 K studies, such as *AHRR*, *F2RL3* and *PRSS23*.^8–11^

Of the 76 DNA methylation sites, 35 were exclusive to the MethylationEPIC BeadChip. Of the 35 sites, 19 confirmed previous smoking loci and 16 were novel signals. Three of the novel sites were annotated to the following genes: *NBR1*, *SLAMF7* and *HERC2*. The protein encoded by *NBR1* functions as a specific autophagy receptor^31^ and is associated with bilateral breast and ovarian cancers. *SLAMF7* encodes a self-ligand receptor of the signalling lymphocytic activation molecule (SLAM) family. Activated SLAM receptors are involved in the regulation of both innate and adaptive immune response.^32^*HERC2* encodes a group of large proteins that are involved in neurodevelopment, DNA damage repair and immune response.^33^ In line with our findings from the blood samples, DNA hypomethylation was also identified at cg05086879 (*MGAT3*) and cg12956751(*ALPP*) in saliva of current smokers in a previous study using the MethylationEPIC BeadChip,^34^ and hypomethylation at cg24797066 was observed to be related to smoking in bronchoalveolar lavage cells.^35^

Our study suggested that smoking had a causal effect on DNA methylation in the blood, which is consistent with the findings from a recent study.^36^ Although our genetic instrument for smoking was weak, the correlation of estimates derived from the MR and EWAS analyses was moderately high. Our results did not support a causal effect of smoking-related DNA methylation in *AHRR*, *F2RL3* and *PRSS23* on the risk of lung cancer, which confirmed and extended the results from a recent MR study.^14^ The 13 DNA methylation sites that were tested for causal relationship with lung cancer risk in our study included seven sites (three novel) from the EPIC BeadChip and six from the 450 K array, whereas the aforementioned MR study^14^ included 16 DNA methylation sites from the 450 K array among which only cg05575921 overlapped with ours. DNA methylation at cg05575921 in *AHRR* has been found to be most strongly influenced by smoking in the current and previous studies.^5^^,^^10^^,^^14^^,^^21^ However, there was no clear evidence for a causal link between DNA methylation at cg05575921 and the risk of lung cancer in our second-step MR analysis nor in the referred MR study.^14^ This is in contrast to previous findings by Fasanelli *et al.,* who reported that hypomethylation of DNA methylation sites in *AHRR* and *F2RL3* may mediate the effect of tobacco smoking on lung cancer risk, based on observational mediation analyses.^5^ Our results indicate this might have been due to residual confounding in the previous mediation analysis. We also identified several other potential mediating DNA methylation sites near or in genes such as *RARA*, *GPR15*, *SLAMF7* and *MIR4505/ELMSAN1.* Among these genes, *SLAMF7* is a novel signal identified by the EPIC array in our study. Our second-step MR analysis, however, did not show evidence for a causal effect of cg19572487 in *RARA*, cg19859270 in *GPR15* or cg25845814 in *MIR4505/ELMSAN1* on the risk of lung cancer. Nor did we find that cg00045592 in *SLAMF7* was causally associated with lung cancer risk.

### Strengths and limitations

There are several strengths to our study. We used the latest Illumina HumanMethylation EPIC BeadChip to analyse DNA methylation, which covers over 850 K DNA methylation sites and thus provides a higher coverage compared with the previous arrays. Blood samples used to generate DNA methylation profiles were collected years before the diagnosis of lung cancer. In HUNT2 this was on average 15 years before diagnosis and therefore reverse causation was unlikely. The information on smoking status and pack-years was recorded years before the diagnosis, which reduced the recall bias. A detailed smoking phenotype was derived based on both smoking quantity in total and smoking status. By using the detailed smoking phenotype, a clear dose-response association of smoking with DNA methylation was demonstrated. To date, there have been few studies investigating the association between smoking and DNA methylation over time using repeated measurements.^10^ Our study showed that smoking-related DNA methylation was reliable: among the 76 DNA methylation sites identified from the EWAS, 75 sites were confirmed in the analysis using repeatedly measured DNA methylation data. In addition, we applied two-step MR analyses to evaluate if causal associations existed between smoking and DNA methylation as well as between DNA methylation and lung cancer risk. Our study confirmed and extended the findings of the previous studies assessing the above causal relationships respectively^14^^,^^36^ by including more and novel methylation sites identified with the EPIC BeadChip.

Our study also has limitations. We used the beta-values of DNA methylation for EWAS as they have intuitively biological interpretation. However, beta-values have severe heteroscedasticity outside the middle methylation range.^37^^,^^38^ The beta difference directly obtained from the beta-value linear regression model can give biased results when beta-values are not between 0.2 and 0.8.^38^ Our study may not have sufficient power to detect a small effect of DNA methylation on the risk of lung cancer. This power issue is reflected by the relatively wider 95% confidence intervals (CIs) in Table 5. Some of the null associations may be due to weak instrument bias, as the mQTLs explained only 0.6% to 6.8% of the variance in DNA methylation on the 13 CpG sites (the putative mediator sites). In two-sample MR, weak instrument bias inclines the association towards the null.^39^ Due to the small number of cases, we were not able to evaluate the causal effect of smoking-related DNA methylation in blood on the risk of specific histological types. Future studies are warranted to investigate the potential causal effect of DNA methylation in blood on risk of lung cancer histological types.

## Conclusion

In conclusion, we identified 16 novel DNA methylation sites related to smoking, using the latest DNA methylation array. Smoking had a causal association with DNA methylation modifications. We did not find evidence for DNA methylation in blood being a causal factor for lung cancer risk. However, the newly identified smoking-related DNA methylation signals have the potential to be explored as additional markers for smoking, to improve the early prediction of lung cancer risk in future studies.

## Supplementary Data

Supplementary data are available at *IJE* online.

## Funding

This work was supported by the Norwegian Cancer Society (project ID 182688–2016) and the Research Council of Norway ‘Gaveforsterkning’. Y.Q.S. was supported by a Researcher grant from the Liaison Committee for education, research and innovation in Central Norway (project ID 2018/42794). R.C.R. is a de Pass VC Research Fellow at the University of Bristol. T.B. is funded by a Wellcome Trust PhD studentship (203746). A.D.B. would like to acknowledge funding from the Wellcome PhD training fellowship for clinicians (204979/Z/16/Z), the Edinburgh Clinical Academic Track (ECAT). R.C.R. and C.L.R. are supported by a Cancer Research UK programme grant (C18281/A19169) and R.C.R., J.L.M., M.S., C.L.R. and T.B. are all members of the MRC Integrative Epidemiology Unit at the University of Bristol supported by the UK Medical Research Council (MC_UU00011/5). The funders had no role in study design, data collection and analysis, decision to publish or preparation of the manuscript. The corresponding authors had access to all the data in the study and had final responsibility for the decision to submit for publication. Where authors are identified as personnel of the International Agency for Research on Cancer/World Health Organization, the authors alone are responsible for the views expressed in this article and they do not necessarily represent the decisions, policy or views of the International Agency for Research on Cancer/World Health Organization.

## Supplementary Material

dyab044_Supplementary_Data

## Data Availability

Data from the HUNT Study that are used in research projects will, when reasonably requested by others, be made available on request to the HUNT Data Access Committee [hunt@medisin.ntnu.no]. The HUNT data access information describes the policy regarding data availability [https://www.ntnu.edu/hunt/data].

## References

[1] GLOBOCAN 2012. Estimated Cancer Incidence, Mortality and Prevalence Worldwide in 2012. v1.0... Lyon, France: IARC, 2012.

[2] Gould MK , Donington J, Lynch WR et al Evaluation of individuals with pulmonary nodules: when is it lung cancer? Diagnosis and management of lung cancer, 3rd ed. American College of Chest Physicians Evidence-Based Clinical Practice Guidelines. Chest 2013;143:e93S–e120S. 23649456 10.1378/chest.12-2351 PMC3749714

[3] Relton CL , Davey Smith G. Is epidemiology ready for epigenetics? Int J Epidemiol 2012;41:5–9. 22422447 10.1093/ije/dys006 PMC3304535

[4] Berdasco M , Esteller M. Aberrant epigenetic landscape in cancer: how cellular identity goes awry. Dev Cell 2010;19:698–711. 21074720 10.1016/j.devcel.2010.10.005

[5] Fasanelli F , Baglietto L, Ponzi E et al Hypomethylation of smoking-related genes is associated with future lung cancer in four prospective cohorts. Nat Commun 2015;6:10192. 26667048 10.1038/ncomms10192 PMC4682166

[6] Nebbioso A , Tambaro FP, Dell’Aversana C, Altucci L. Cancer epigenetics: Moving forward. PLoS Genet 2018;14:e1007362. 29879107 10.1371/journal.pgen.1007362 PMC5991666

[7] Herceg Z , Ghantous A, Wild CP et al Roadmap for investigating epigenome deregulation and environmental origins of cancer. Int J Cancer 2018;142:874–82. 28836271 10.1002/ijc.31014 PMC6027626

[8] Ambatipudi S , Cuenin C, Hernandez-Vargas H et al Tobacco smoking-associated genome-wide DNA methylation changes in the EPIC study. Epigenomics 2016;8:599–618. 26864933 10.2217/epi-2016-0001

[9] Joehanes R , Just AC, Marioni RE et al Epigenetic signatures of cigarette smoking. Circ Cardiovasc Genet 2016;9:436–47. 27651444 10.1161/CIRCGENETICS.116.001506 PMC5267325

[10] Wilson R , Wahl S, Pfeiffer L et al The dynamics of smoking-related disturbed methylation: a two time-point study of methylation change in smokers, non-smokers and former smokers. BMC Genomics 2017;18:805. 29047347 10.1186/s12864-017-4198-0 PMC6389045

[11] Zeilinger S , Kuhnel B, Klopp N et al Tobacco smoking leads to extensive genome-wide changes in DNA methylation. PLoS One 2013;8:e63812. 23691101 10.1371/journal.pone.0063812 PMC3656907

[12] Stueve TR , Li WQ, Shi J et al Epigenome-wide analysis of DNA methylation in lung tissue shows concordance with blood studies and identifies tobacco smoke-inducible enhancers. Hum Mol Genet 2017;26:3014–27. 28854564 10.1093/hmg/ddx188 PMC5886283

[13] Lee PN , Forey BA, Coombs KJ. Systematic review with meta-analysis of the epidemiological evidence in the 1900s relating smoking to lung cancer. BMC Cancer 2012;12:385. 22943444 10.1186/1471-2407-12-385 PMC3505152

[14] Battram T , Richmond RC, Baglietto L et al Appraising the causal relevance of DNA methylation for risk of lung cancer. Int J Epidemiol 2019;48:1493–504. 31549173 10.1093/ije/dyz190 PMC6857764

[15] Davey Smith G , Hemani G. Mendelian randomization: genetic anchors for causal inference in epidemiological studies. Hum Mol Genet 2014;23:R89–98. 25064373 10.1093/hmg/ddu328 PMC4170722

[16] Davies NM , Holmes MV, Davey Smith G. Reading Mendelian randomisation studies: a guide, glossary, and checklist for clinicians. BMJ 2018;362:k601. 30002074 10.1136/bmj.k601 PMC6041728

[17] Bell JT , Pai AA, Pickrell JK et al DNA methylation patterns associate with genetic and gene expression variation in HapMap cell lines. Genome Biol 2011;12:405. R10. 10.1186/gb-2011-12-1-r10 PMC3091299 21251332

[18] Hannon E , Gorrie-Stone TJ, Smart MC et al Leveraging DNA-methylation quantitative-trait loci to characterize the relationship between methylomic variation, gene expression, and complex traits. Am J Hum Genet 2018;103:654–65. 30401456 10.1016/j.ajhg.2018.09.007 PMC6217758

[19] Langdon R , Richmond R, Elliott HR et al Identifying epigenetic biomarkers of established prognostic factors and survival in a clinical cohort of individuals with oropharyngeal cancer. Clin Epigenet 2020;12:95. 10.1186/s13148-020-00870-0 PMC7322918 32600451

[20] Relton CL , Davey Smith G. Two-step epigenetic Mendelian randomization: a strategy for establishing the causal role of epigenetic processes in pathways to disease. Int J Epidemiol 2012;41:161–76. 22422451 10.1093/ije/dyr233 PMC3304531

[21] Baglietto L , Ponzi E, Haycock P et al DNA methylation changes measured in pre-diagnostic peripheral blood samples are associated with smoking and lung cancer risk. Int J Cancer 2017;140:50–61. 27632354 10.1002/ijc.30431 PMC5731426

[22] Sandanger TM , Nøst TH, Guida F et al DNA methylation and associated gene expression in blood prior to lung cancer diagnosis in the Norwegian Women and Cancer cohort. Sci Rep 2018;8:16714. 30425263 10.1038/s41598-018-34334-6 PMC6233189

[23] Krokstad S , Langhammer A, Hveem K et al Cohort Profile: The HUNT study. Norway Int J Epidemiol 2013;42:968–77. 22879362 10.1093/ije/dys095

[24] Sun YQ , Brumpton BM, Bonilla C et al Serum 25-hydroxyvitamin D levels and risk of lung cancer and histologic types: a Mendelian randomisation analysis of the HUNT study. Eur Respir J 2018*;* 51*:* 18000329. 10.1183/13993003.00329-2018 PMC7614587 29748306

[25] Leek JT , Storey JD. Capturing heterogeneity in gene expression studies by surrogate variable analysis. PLoS Genet 2007;3:e161–35. 10.1371/journal.pgen.0030161 PMC1994707 17907809

[26] Min JL , Hemani G, Davey Smith G, Relton C, Suderman M. Meffil: efficient normalization and analysis of very large DNA methylation datasets. Bioinformatics 2018;34:3983–89. 29931280 10.1093/bioinformatics/bty476 PMC6247925

[27] Staley JR , Suderman M, Simpkin AJ et al Longitudinal analysis strategies for modelling epigenetic trajectories. Int J Epidemiol 2018;47:516–25. 29462323 10.1093/ije/dyy012 PMC5913606

[28] Tobacco and Genetics Consortium. Genome-wide meta-analyses identify multiple loci associated with smoking behavior. Nat Genet 2010;42:441–47. 20418890 10.1038/ng.571 PMC2914600

[29] Smith BH , Campbell A, Linksted P et al Cohort Profile: Generation Scotland: Scottish Family Health Study (GS:SFHS). The study, its participants and their potential for genetic research on health and illness. Int J Epidemiol 2013;42:689–700. 22786799 10.1093/ije/dys084

[30] McKay JD , Hung RJ, Han Y et al; SpiroMeta Consortium. Large-scale association analysis identifies new lung cancer susceptibility loci and heterogeneity in genetic susceptibility across histological subtypes. Nat Genet 2017;49:1126–32. 28604730 10.1038/ng.3892 PMC5510465

[31] Kirkin V , Lamark T, Sou YS et al A role for NBR1 in autophagosomal degradation of ubiquitinated substrates. Mol Cell 2009;33:505–16. 19250911 10.1016/j.molcel.2009.01.020

[32] Chen J , Zhong MC, Guo H et al SLAMF7 is critical for phagocytosis of haematopoietic tumour cells via Mac-1 integrin. Nature 2017;544:493–97. 28424516 10.1038/nature22076 PMC5565268

[33] Sánchez-Tena S , Cubillos-Rojas M, Schneider T, Rosa JL. Functional and pathological relevance of HERC family proteins: a decade later. Cell Mol Life Sci 2016;73:1955–68. 26801221 10.1007/s00018-016-2139-8 PMC11108380

[34] Barcelona V , Huang Y, Brown K et al Novel DNA methylation sites associated with cigarette smoking among African Americans. Epigenetics 2019;14:383–91. 30915882 10.1080/15592294.2019.1588683 PMC6557550

[35] Ringh MV , Hagemann-Jensen M, Needhamsen M et al Tobacco smoking induces changes in true DNA methylation, hydroxymethylation and gene expression in bronchoalveolar lavage cells. EBioMedicine 2019;46:290–304. 31303497 10.1016/j.ebiom.2019.07.006 PMC6710853

[36] Li S , Wong EM, Bui M et al Causal effect of smoking on DNA methylation in peripheral blood: a twin and family study. Clin Epigenet 2018;10:18. 10.1186/s13148-018-0452-9 PMC5810186 29456763

[37] Du P , Zhang X, Huang CC et al Comparison of Beta-value and M-value methods for quantifying methylation levels by microarray analysis. BMC Bioinformatics 2010;11:587. 21118553 10.1186/1471-2105-11-587 PMC3012676

[38] Xie C , Leung YK, Chen A, Long DX, Hoyo C, Ho SM. Differential methylation values in differential methylation analysis. Bioinformatics 2019;35:1094–97. 30184051 10.1093/bioinformatics/bty778 PMC6449748

[39] Lawlor DA. Commentary: Two-sample Mendelian randomization: opportunities and challenges. Int J Epidemiol 2016;45:908–15. 27427429 10.1093/ije/dyw127 PMC5005949

