# Supplementary material for: Assessing the role of genome-wide DNA methylation between smoking and risk of lung cancer using repeated measurements: the HUNT study
Source: Int J Epidemiol. 2021 Mar 17;50(5):1482–97. doi: 10.1093/ije/dyab044 (PMC8580278; doi:10.1093/ije/dyab044)

Online Supplementary Materials

Assessing the role of genome-wide DNA methylation between smoking and risk of lung cancer using repeated measurements: the HUNT Study

Yi-Qian Sun, Rebecca C Richmond, Matthew Suderman, Josine L Min, Thomas Battram, Arnar Flatberg, Vidar Beisvag, Therese Haugdahl Nøst, Florence Guida, Lin Jiang, Sissel Gyrid Freim Wahl, Arnulf Langhammer, Frank Skorpen, Rosie M Walker, Andrew D Bretherick, Yanni Zeng, Yue Chen, Mattias Johansson, Torkjel M Sandanger, Caroline L Relton, Xiao-Mei Mai

SUPPLEMENTARY TEXT

Quality control (QC) and functional normalization of the DNA methylation data

The quality control (QC) processing step using raw data files (IDAT) included background correction, dye bias correction, sex prediction and cell count estimation. Background and dye bias correction were performed to enhance the signal-to-noise-ratio of the methylation array. Predicted sex was used to detect sample mix-ups. The cell count estimation (B cell, $CD4^+$ cells, $CD8^+$ cells, eosinophil, monocytes, neutrophils and natural killer cells) was obtained using the Houseman algorithm [1]. Among the 560 samples, we removed 6 sex detection outliers, 1 sex detection mismatch and 11 outliers from the methylated and unmethylated signal comparison. The 11 outliers were samples with predicted median methylated signal being more than 3 standard deviations from the expected signal. After removal of these 18 samples, the QC plot depicting the median intensity methylated vs. unmethylated signal shows a random distribution of the samples (Supplementary Figure S1). Functional normalization was undertaken on the remaining samples to remove technical batch effects. The functional normalization algorithm developed by Fortin *et al.* [2] used the internal control probes present on the array to infer between-array technical variation. Principal components were applied to remove batch effects further. The QC and normalization of DNA methylation data were performed with R package *meffil* (version 1.1.0) [3].

Statistical analysis

All statistical analyses were performed with R (version 3.6.1) or Stata/SE 15.1 (StataCorp, College Station, TX). Different sets of data that were used for specific statistical analyses are described in Supplementary Table S1.

EWAS for smoking in controls (cross-sectional analysis)

We first performed an EWAS for the smoking phenotype (the 7 levels) in blood samples collected from the controls in HUNT2. Linear regressions were performed with DNA methylation beta-values as the outcome and smoking phenotype as the exposure. Covariates were included in regression models to adjust for effects of sex, age, and estimated cell counts. Surrogate variable analysis (SVA) [4] was used to generate 12 variables which were included as covariates in EWAS models in order to adjust for batch and other technical artifacts. The *P* value cut-off was set at epigenome-wide significance level (5×10^{-8}). EWAS for smoking was performed with R package *meffil* (version 1.1.0) [3]. Genomic regions were annotated using Human MethylationEPIC probe annotations (the positions are based on human genome reference build b37). The following annotations were used as locations in relation to gene

region: TSS1500 (200 to 1500 nucleotides upstream of transcription start site); TSS200 (up to 200 nucleotides upstream of transcription start site); 5' UTR (5' untranslated region); 1st Exon; Body (gene body); and 3' UTR (3' untranslated region).

Confirmation of EWAS for smoking

To confirm the associations identified form the EWAS for smoking, we performed an analysis using repeatedly measured DNA methylation data from both the HUNT2 and the HUNT3 samples in relation to the smoking phenotype in HUNT2 among the controls. We used a less computationally intensive strategy by performing linear regression across the repeated measures with cluster-robust standard errors (LMRSE) [5]. Sex, age and estimated cell counts in HUNT2 were included as covariates in the analysis and Bonferroni correction was applied for the epigenome-wide significant DNA methylation sites identified from the EWAS. In order to confirm the results from the LMRSE, computationally intensive linear mixed effects model (LMEM) with random intercept was also performed for randomly selected 1000 DNA methylation sites across the entire genome using the repeatedly measured DNA methylation data. The analyses of LMRSE and LMEM were performed with R packages *lmrse* (version 2.0), *lme4* (version 1.1.21) and *omics* (version 0.1.5), respectively.

Effect of change in smoking on change in DNA methylation in controls

We also explored the possible effect of change in smoking status between HUNT2 and HUNT3 (categorized as decrease, no change or increase) on change in DNA methylation (beta-value of DNA methylation in HUNT3 minus beta-value of DNA methylation in HUNT2) among the controls. Sex, age and cell count estimates (both HUNT2 and HUNT3) were included as covariates in the model. Bonferroni correction was applied for the epigenome-wide significant DNA methylation sites identified from the EWAS for smoking. The association between changes in DNA methylation and changes in smoking status was tested using linear regression models using R package *meffil* (version 1.1.0) [3].

EWAS for lung cancer (case-control analysis)

EWAS for lung cancer was performed among the lung cancer cases vs. controls with DNA methylation measured in HUNT2 and HUNT3 respectively as the exposure. Covariates included smoking phenotype, sex, age and cell count estimates and the epigenome-wide *P*-value cut-off was set at 5×10^{-8}. Logistic regression analyses were performed in R package *ewaff* (version 0.0.1) (https://github.com/perishky/ewaff).

Mediation analysis of DNA methylation on the pathway between smoking and risk of lung cancer

The smoking-related DNA methylation sites that overlapped between the EWAS for smoking and the EWAS for lung cancer in the HUNT2 samples were individually evaluated as potential mediators between the smoking phenotype and lung cancer using mediation analysis. Sex, age and cell count estimates were included as covariates in the model. A counterfactual framework was applied, and the mediation analysis was performed with R package *mma* (version 9.0.0) [6]. Multiple mediators were then considered simultaneously, and a weighted methylation score was calculated as the sum of methylation beta-value at each mediator site weighted by its effect size on lung cancer. The indirect effect carried by the individual mediator or by the weighted methylation score was separated from the total effect of smoking phenotype.

Two-step Mendelian randomization analyses

MR may be used to infer causal effect of the exposure if genetic variants used as instrumental variables satisfy the following three fundamental assumptions: 1) strongly associated with the exposure; 2) independent of confounding factors of the observational association; and 3) associated with the outcome only through the exposure (no horizontal pleiotropy) [7, 8].

Potential causal associations were tested by applying MR two times, hence it is called two-step MR. A first step MR analysis was applied to evaluate the causal effect of smoking on DNA methylation. We used a smoking genetic score including 3 SNPs as an instrumental variable for the smoking phenotype (7 levels): rs6265 (*BDNF*) associated with smoking initiation, rs1051730 (*CHRNA3*) with smoking quantity, and rs3025343 (*DBH*) with smoking cessation [9]. The genetic score was calculated by summing up the risk alleles (associated with starting smoking, smoking more, and not quitting smoking) of these 3 SNPs for each individual. One-sample MR using the two-stage least square (2SLS) method was applied to investigate a causal relationship between smoking and DNA methylation at the sites identified in the EWAS for smoking. Sex, age and predicted cell counts were included in the MR models. Pearson correlation coefficient was used to evaluate the relationship between estimates derived from the MR and EWAS analyses.

A second step MR was performed to evaluate the putative causal association between DNA methylation and the risk of lung cancer. We applied a two-sample MR in order to leverage power from large genome-wide association studies (GWAS). Instruments for the DNA methylation sites detected as putative mediators with the mediation analyses were

extracted from an mQTL GWAS in Generation Scotland. Generation Scotland has been described elsewhere [10, 11]. In brief, a subset of participants (n=5 101) from the Generation Scotland underwent DNA methylation profiling by Infinium EPIC array. After QC and normalization, DNA methylation M-values at 638 737 DNA methylation sites were tested for associations with genetic variants to identify mQTLs. Genetic variants were identified as mQTLs (both *cis* and *trans*) if they were associated with a DNA methylation site ($P<1.0\times10^{-7}$). Independent associations were identified by applying linkage disequilibrium (LD) clumping with an R^2 of 0.01. Summary statistics of lung cancer GWAS were derived from McKay *et al*. [12] with sample size 85 716 (cases 29 266 vs. controls 56 450). The inverse-variance weighted (IVW) method or Wald ratio method (when only one mQTL as instrumental variable) as implemented in the R package *TwoSampleMR* (version 0.4.25) was used to calculate the causal estimates. Pearson correlation coefficient was used to express the strength of the relationship between the estimates derived from the two-sample MR and the EWAS for lung cancer in HUNT2.

References

1. Houseman EA, Accomando WP, Koestler DC, et al. DNA methylation arrays as surrogate measures of cell mixture distribution. *BMC Bioinformatics* 2012; **13**: 86-.
2. Fortin J-P, Labbe A, Lemire M, et al. Functional normalization of 450k methylation array data improves replication in large cancer studies. *Genome Biol* 2014; **15**: 503-.
3. Min JL, Hemani G, Davey Smith G, Relton C, Suderman M. Meffil: efficient normalization and analysis of very large DNA methylation datasets. *Bioinformatics (Oxford, England)* 2018; **34**: 3983-9.
4. Leek JT, Storey JD. Capturing heterogeneity in gene expression studies by surrogate variable analysis. *PLoS genetics* 2007; **3**: 1724-35.
5. Staley JR, Suderman M, Simpkin AJ, et al. Longitudinal analysis strategies for modelling epigenetic trajectories. *Int J Epidemiol* 2018; **47**: 516-25.
6. Yu Q, Wu X, Li B, Scribner RA. Multiple mediation analysis with survival outcomes: With an application to explore racial disparity in breast cancer survival. *Stat Med* 2019; **38**: 398-412.
7. Davey Smith G, Hemani G. Mendelian randomization: genetic anchors for causal inference in epidemiological studies. *Hum Mol Genet* 2014; **23**: R89-98.
8. Davies NM, Holmes MV, Davey Smith G. Reading Mendelian randomisation studies: a guide, glossary, and checklist for clinicians. *BMJ* 2018; **362**: k601.
9. Tobacco, Genetics C. Genome-wide meta-analyses identify multiple loci associated with smoking behavior. *Nat Genet* 2010; **42**: 441-7.
10. Smith BH, Campbell A, Linksted P, et al. Cohort Profile: Generation Scotland: Scottish Family Health Study (GS:SFHS). The study, its participants and their potential for genetic research on health and illness. *International journal of epidemiology* 2013; **42**: 689-700.

11. Langdon R, Richmond R, Elliott HR, et al. Identifying epigenetic biomarkers of established prognostic factors and survival in a clinical cohort of individuals with oropharyngeal cancer. *Clin Epigenetics* 2020; **12**: 95.
12. McKay JD, Hung RJ, Han Y, et al. Large-scale association analysis identifies new lung cancer susceptibility loci and heterogeneity in genetic susceptibility across histological subtypes. *Nat Genet* 2017; **49**: 1126-32.

Supplementary Table S1. Statistical analyses were performed using different sets of data

	HUNT2		HUNT3	
	Lung cancer cases	Controls	Lung cancer cases	Controls
EWAS for smoking in controls (cross-sectional analysis)		√		
Confirmation of EWAS for smoking using repeatedly measured genome-wide DNA methylation in controls		√		√
Effect of change in smoking on change in DNA methylation in controls		√		√
EWAS for lung cancer in cases vs. controls	√		√	
Mediation analysis of DNA methylation on the pathway between smoking and risk of lung cancer	√			
First step MR analyses		√		
Second step MR analyses	This two-sample MR used summary statistics of GWAS			

EWAS: epigenome-wide association study; GWAS: genome-wide association study; MR: Mendelian randomization

√: the dataset was used.

Supplementary Table S2. Distributions of baseline characteristics in incident lung cancer cases and controls in a nested case-control design of the HUNT Study

	Lung cancer cases	Controls
**HUNT2 study (1995–97)**		
No. of participants (n=276)	139	137
Age (years)	56.4±8.1	55.6±8.4
Sex		
Women	62 (44.6%)	62 (45.3%)
Men	77 (55.4%)	75 (54.7%)
Smoking status		
Never smokers	10 (7.2%)	66 (48.2%)
Former smokers	20 (14.4%)	38 (27.7%)
Current smokers	106 (76.3%)	30 (21.9%)
Unknown*	3 (2.2%)	3 (2.2%)
Smoking phenotype		
0: Never smokers	10 (7.2%)	66 (48.2%)
1: Former smokers ≤10.0 pyrs	3 (2.2%)	21 (15.3%)
2: Former smokers 10.1–20.0 pyrs	3 (2.2%)	7 (5.1%)
3: Former smokers ≥20.1 pyrs	11 (7.9%)	4 (2.9%)
4: Current smokers ≤10.0 pyrs	9 (6.5%)	7 (5.1%)
5: Current smokers 10.1–20.0 pyrs	36 (25.9%)	13 (9.5%)
6: Current smokers ≥20.1 pyrs	55 (39.6%)	10 (7.3%)
7: Unknown pyrs*	12 (8.6%)	9 (6.6%)
**HUNT3 study (2006–08)**		
No. of participants (n=266)	131	135
Age (years)	67.8±8.2	66.8±8.5
Sex		
Women	61 (46.6%)	63 (46.7%)
Men	70 (53.4%)	72 (53.3%)
Smoking status		
Never smokers	6 (4.6%)	60 (44.4%)
Former smokers	45 (34.4%)	49 (36.3%)
Current smokers	72 (55.0%)	24 (17.8%)
Unknown*	8 (6.1%)	2 (1.5%)
Smoking phenotype		
0: Never smokers	6 (4.6%)	60 (44.4%)
1: Former smokers ≤10.0 pyrs	3 (2.3%)	16 (11.9%)
2: Former smokers 10.1–20.0 pyrs	5 (3.8%)	11 (8.2%)
3: Former smokers ≥20.1 pyrs	28 (21.4%)	9 (6.7%)

4: Current smokers ≤10.0 pyrs	2 (1.5%)	7 (5.2%)
5: Current smokers 10.1–20.0 pyrs	9 (6.9%)	7 (5.2%)
6: Current smokers ≥20.1 pyrs	48 (36.6%)	9 (6.7%)
7: Unknown pyrs*	30 (22.9%)	16 (11.9%)

pyrs: pack-years of active smoking; data are given as number of participants (percentage) or mean ± standard deviation

*excluded from the analyses

Supplementary Table S3. Mendelian randomization analysis of associations between smoking and DNA methylation for the 76 smoking-related DNA methylation sites (n=134)

**DNAm sites**	**Coefficient**	**SE**	**95% CI**		***P* value**
cg05575921	-0.061	0.035	-0.129	0.007	0.080
cg03636183	-0.017	0.020	-0.057	0.023	0.406
cg21566642	-0.036	0.024	-0.084	0.011	0.133
cg17739917	-0.008	0.017	-0.041	0.025	0.631
cg01940273	-0.010	0.018	-0.045	0.025	0.559
cg21911711	0.000	0.012	-0.025	0.024	0.981
cg05086879	-0.013	0.012	-0.037	0.012	0.301
cg26703534	-0.014	0.011	-0.037	0.009	0.221
cg21161138	-0.024	0.013	-0.050	0.002	0.070
cg17087741	-0.012	0.011	-0.034	0.010	0.287
cg04180924	-0.002	0.004	-0.010	0.007	0.707
cg19859270	-0.006	0.005	-0.016	0.004	0.271
**cg14391737**	**-0.058**	**0.020**	**-0.097**	**-0.019**	**0.004**
cg18110140	-0.028	0.015	-0.057	0.001	0.060
cg14466441	-0.004	0.005	-0.014	0.006	0.395
**cg09338374**	**0.020**	**0.008**	**0.004**	**0.037**	**0.018**
cg25648203	-0.010	0.011	-0.033	0.012	0.367
cg05284742	0.001	0.008	-0.015	0.017	0.874
cg07943658	0.010	0.011	-0.011	0.031	0.364
**cg02978227**	**-0.018**	**0.007**	**-0.032**	**-0.005**	**0.009**
cg26768182	-0.014	0.010	-0.033	0.005	0.139
cg03329539	-0.019	0.011	-0.040	0.003	0.087
cg12803068	0.013	0.030	-0.046	0.071	0.670
cg25845814	0.001	0.008	-0.015	0.017	0.924
**cg16841366**	**-0.035**	**0.018**	**-0.070**	**0.000**	**0.049**
cg22812571	-0.023	0.017	-0.056	0.011	0.188
cg19572487	-0.016	0.014	-0.043	0.011	0.237
cg18754985	-0.003	0.005	-0.012	0.006	0.566
cg10765427	-0.011	0.007	-0.025	0.003	0.135
cg24859433	0.002	0.009	-0.016	0.019	0.867
**cg12956751**	**-0.017**	**0.008**	**-0.032**	**-0.002**	**0.028**
cg03384915	-0.006	0.006	-0.018	0.005	0.298
cg05533761	-0.012	0.020	-0.051	0.028	0.562
**cg13849276**	**-0.028**	**0.014**	**-0.056**	**-0.001**	**0.042**
cg21611682	-0.014	0.009	-0.031	0.002	0.092
cg00045592	-0.026	0.013	-0.051	0.000	0.053
**cg00475490**	**-0.031**	**0.011**	**-0.053**	**-0.009**	**0.007**

cg08064403	-0.002	0.006	-0.014	0.010	0.725
cg04180046	0.015	0.019	-0.023	0.053	0.431
cg15342087	-0.001	0.008	-0.018	0.015	0.858
cg13193840	0.002	0.008	-0.014	0.019	0.789
cg05009104	0.008	0.018	-0.028	0.043	0.665
cg19885130	0.001	0.016	-0.030	0.032	0.937
cg09935388	-0.019	0.024	-0.067	0.029	0.430
cg04551776	-0.015	0.009	-0.032	0.003	0.111
cg11660018	-0.013	0.011	-0.034	0.008	0.227
cg23079012	-0.007	0.010	-0.026	0.013	0.495
cg10750182	0.000	0.007	-0.014	0.014	0.971
cg14712058	-0.007	0.008	-0.023	0.009	0.383
**cg22222502**	**-0.030**	**0.012**	**-0.054**	**-0.007**	**0.011**
cg25013095	-0.002	0.002	-0.006	0.003	0.477
cg04956244	0.005	0.006	-0.006	0.017	0.362
**cg14580211**	**-0.032**	**0.012**	**-0.055**	**-0.008**	**0.008**
cg20295214	-0.015	0.008	-0.032	0.002	0.078
cg15417641	-0.001	0.023	-0.046	0.044	0.950
cg01744331	-0.011	0.010	-0.032	0.009	0.260
**cg15212295**	**-0.013**	**0.006**	**-0.025**	**0.000**	**0.045**
cg02657160	-0.006	0.007	-0.020	0.008	0.415
cg00592046	-0.024	0.026	-0.075	0.028	0.366
cg04387347	-0.012	0.015	-0.042	0.018	0.429
cg16758086	-0.002	0.009	-0.019	0.015	0.847
cg14753356	-0.008	0.011	-0.029	0.013	0.462
**cg13258799**	**-0.018**	**0.008**	**-0.034**	**-0.003**	**0.023**
cg14919440	-0.010	0.014	-0.037	0.016	0.448
cg18387338	-0.011	0.008	-0.026	0.004	0.154
cg03528016	0.013	0.008	-0.003	0.030	0.113
cg12876356	-0.018	0.025	-0.067	0.031	0.480
cg06644428	-0.001	0.013	-0.026	0.025	0.960
cg25001882	-0.014	0.009	-0.032	0.004	0.119
cg06035956	-0.005	0.004	-0.014	0.003	0.221
cg24797066	-0.005	0.006	-0.018	0.008	0.452
cg20062762	0.000	0.005	-0.010	0.011	0.949
cg12939236	-0.009	0.009	-0.026	0.007	0.269
cg16508202	0.008	0.005	-0.002	0.018	0.105
cg11554391	-0.013	0.008	-0.029	0.003	0.109
cg19089201	0.003	0.020	-0.036	0.042	0.871

CI: confidence interval; DNAm: DNA methylation; SE: standard error.

DNAm sites with *P* value <0.05 are marked with bold font.

Supplementary Table S4. Second step Mendelian randomization of DNA methylation and risk of lung cancer using *cis* mQTL

DNAm sites as putative mediators	Chr	Gene	Instrumental variable (*cis* mQTL)	Variance explained (%)	OR*	95% CI		*P* value
cg19859270‡	3	*GPR15*	rs1529047	1.8	1.05	0.82	1.36	0.69
cg05575921	5	*AHRR*	rs11956656	0.9	0.96	0.80	1.16	0.70
cg25845814†, ‡	14	*MIR4505; ELMSAN1*	rs111686083	0.4	1.21	0.94	1.57	0.14
cg26768182†	9		rs78581928	0.9	1.23	1.05	1.46	0.01
cg24797066†	20		rs602598	0.6	1.00	0.80	1.25	0.99
cg21911711†	19	*F2RL3*	rs56298289	4.9	0.96	0.89	1.03	0.26
cg00475490†	11	*PRSS23*	rs2279046	3.3	1.05	0.96	1.16	0.25
cg00045592†	1	*SLAMF7*	rs3766373	1.8	0.94	0.82	1.07	0.32
cg03329539	2		rs13023370	4.0	1.01	0.93	1.10	0.85
cg21161138‡	5	*AHRR*	rs2466287	0.4	0.91	0.69	1.20	0.52

Chr: chromosome; DNAm: DNA methylation; mQTL: methylation quantitative trait locus

*Per 1-unit increase of DNA methylation M-value

†Exclusively in EPIC Beadchip

‡$P<1\times10^{-5}$ for association with the mQTLs as no mQTL found if smaller *P*-value was set.

Summary statistics for associations of *cis* mQTLs with lung cancer were not available for 4 putative mediators (cg15342087, cg19572487, cg14391737, and cg24859433).

Supplementary Figure S1. The QC plot shows the median intensity methylated vs. unmethylated signal after the QC preprocessing. The plot is roughly a random cloud with the dots fitting in 3 standard deviations (dotted red lines) from the regression line (solid red line) of median methylated signal vs. median unmethylated signal. QC: quality control.

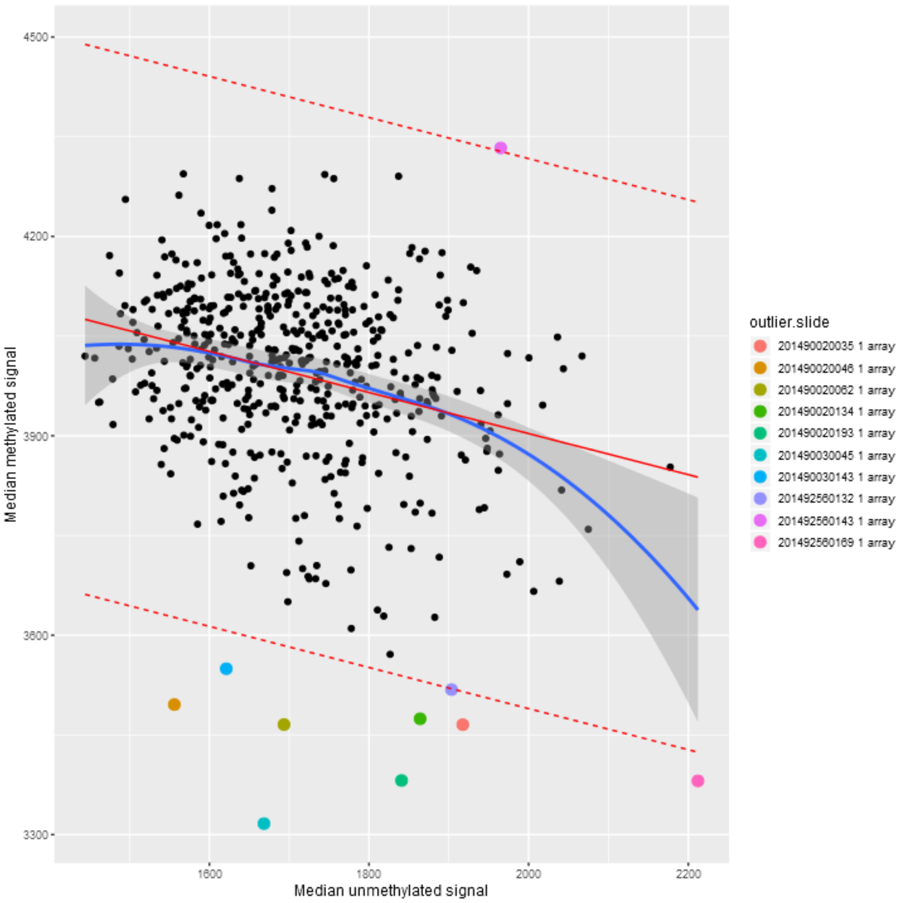

Supplementary Figure S2. Manhattan plot for associations between smoking and genome-wide DNAm in blood samples collected in HUNT2 in controls (n=128). All points above the top red line are at $P < 5x10^{-8}$ and have a positive coefficient (hypermethylation) whereas all points below the bottom red line are at $P < 5x10^{-8}$ and have a negative coefficient (hypomethylation). In total, 76 DNA methylation sites are associated with the smoking phenotype. Chr: chromosome; DNAm: DNA methylation; HUNT: The Trøndelag Health Study.

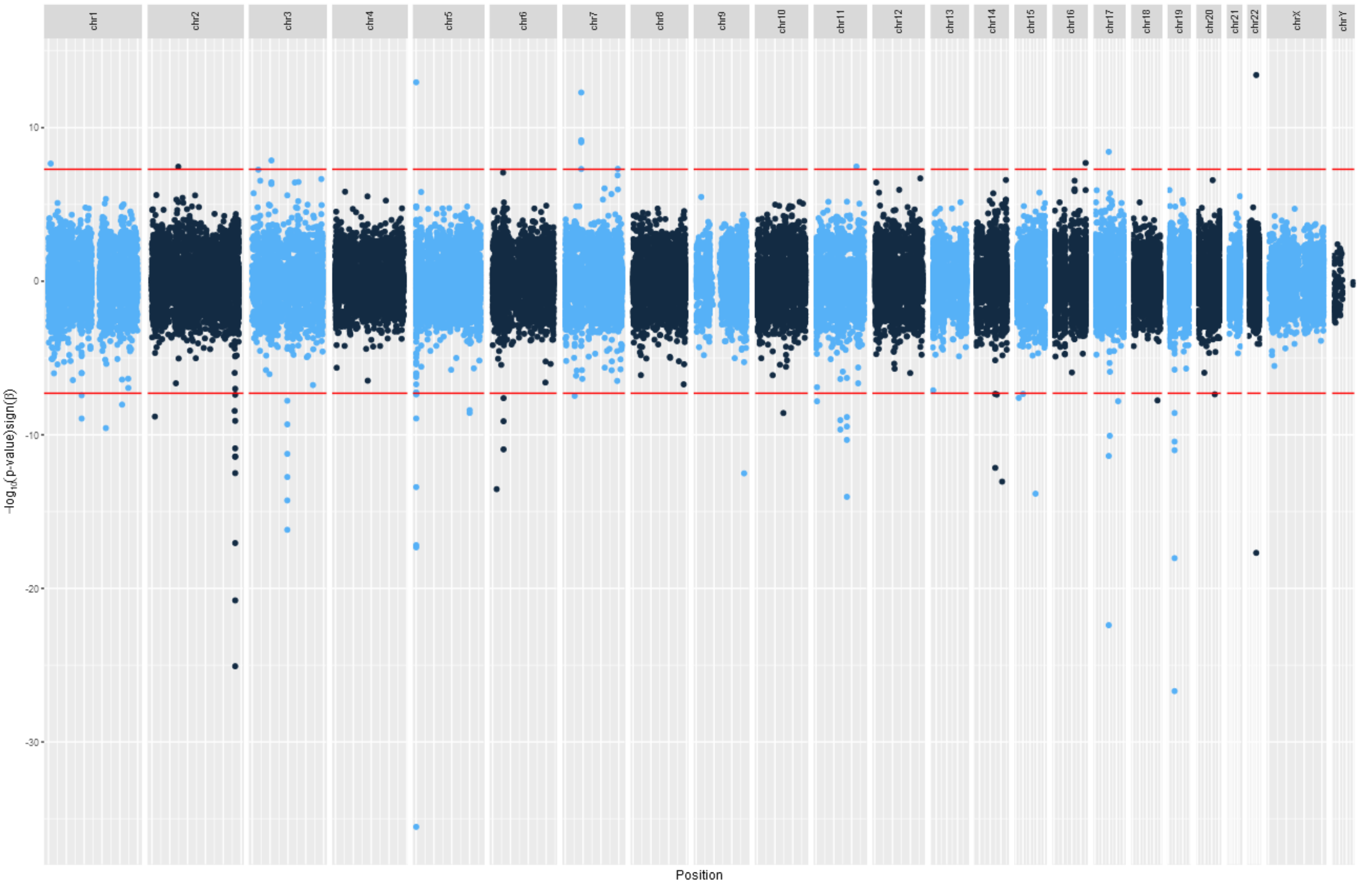

Supplementary Figure S3. Correlation between LMRSE and LMEM estimates for the randomly selected 1000 DNAm site associated with smoking. DNAm: DNA methylation; LMRSE: linear regression model with cluster-robust standard errors; LMEM: linear mixed effects model

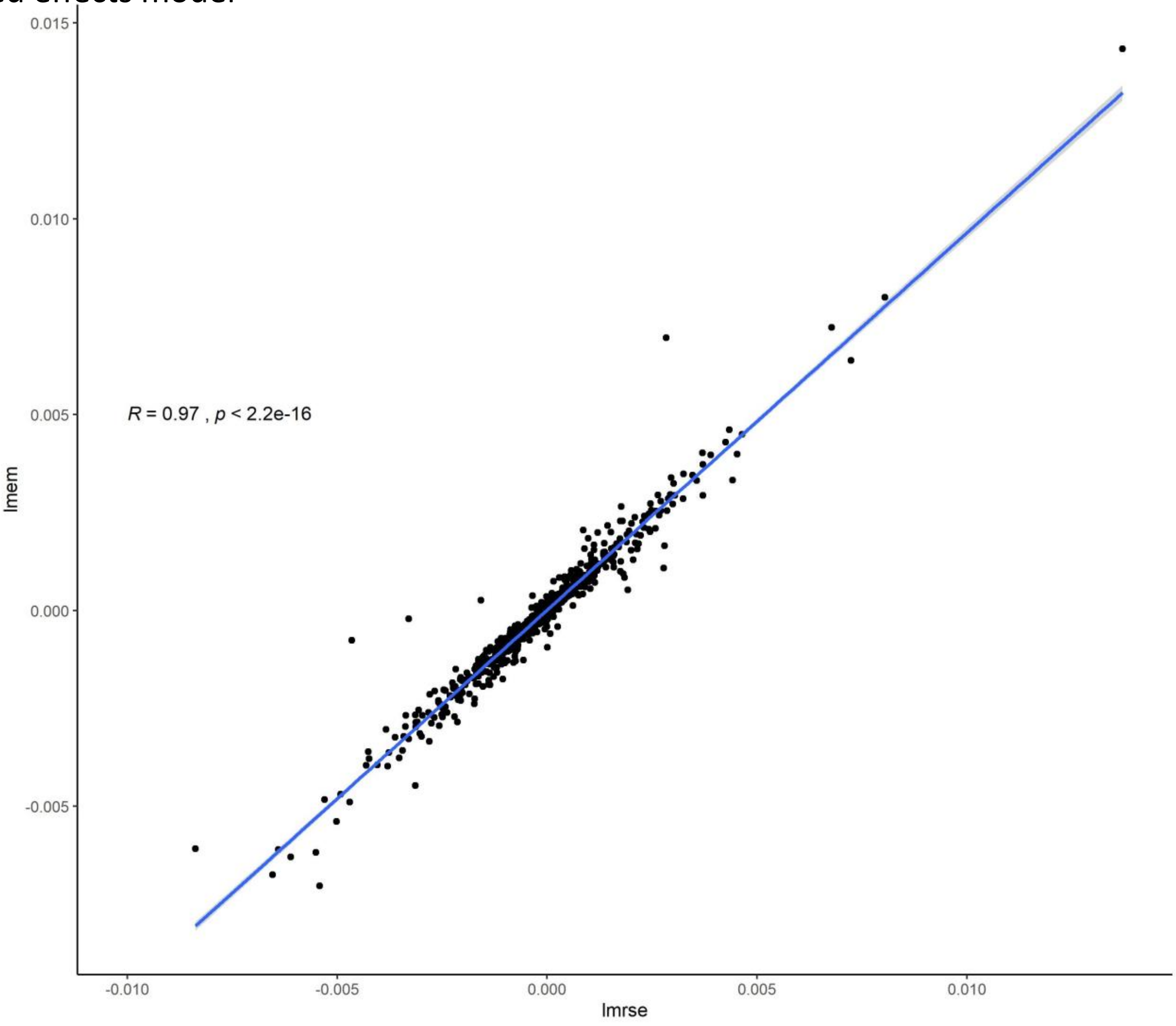

Supplementary Figure S4. Correlation between cross-sectional and longitudinal estimates of smoking-DNA methylation associations for the smoking-related 76 DNAm sites. The longitudinal analysis was performed using repeatedly measured DNAm by linear regression with cluster-robust standard errors (LMRSE).

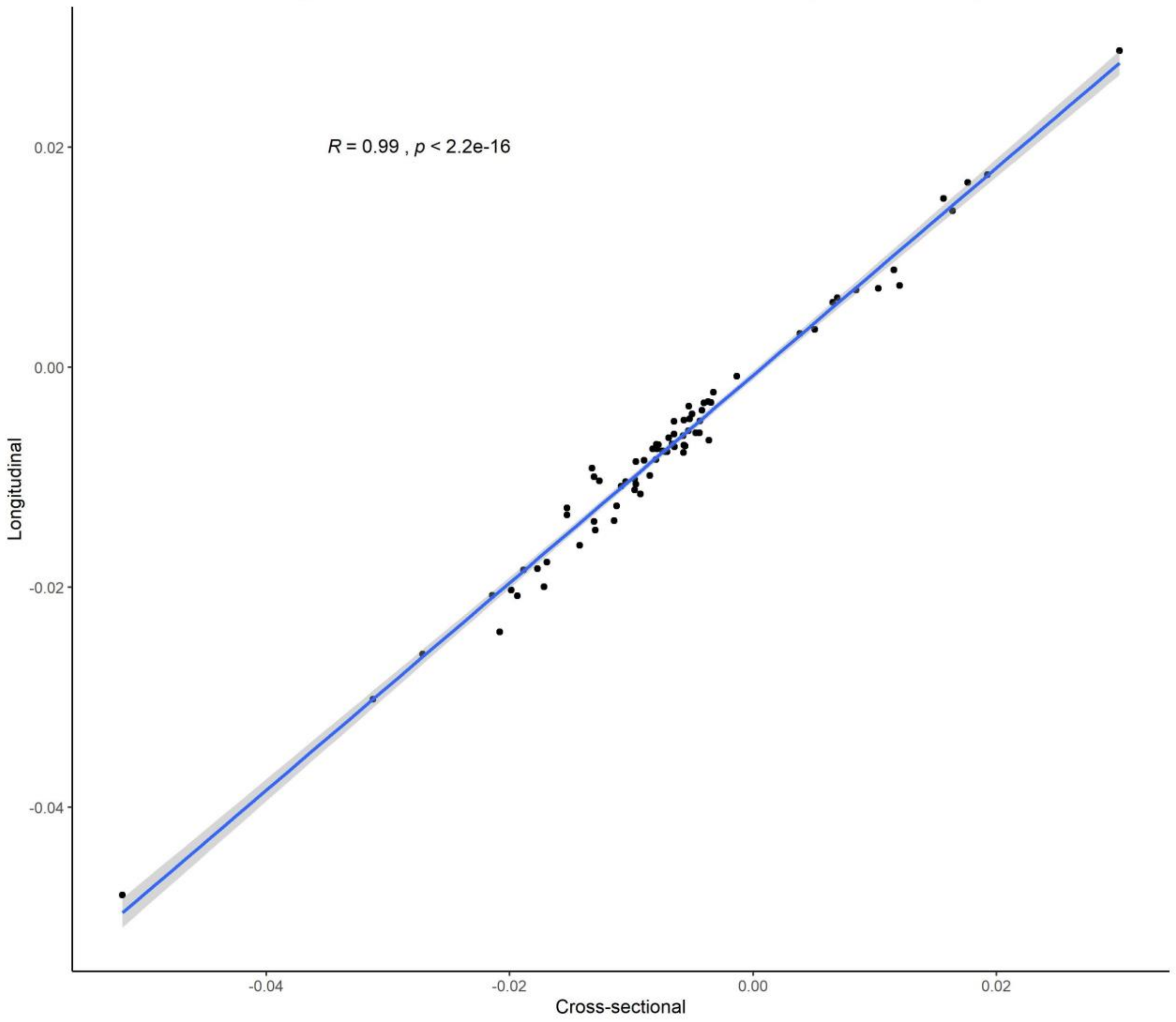

Supplementary Figure S5. Manhattan plot for associations between genome-wide DNAm and risk of lung cancer in blood samples collected in HUNT2 (139 cases vs. 137 controls). All points below the bottom red line are at $P < 5 \times 10^{-8}$ and have a negative coefficient (increase in DNA methylation is associated with decreased risk of lung cancer). In total, 50 DNA methylation sites are associated with risk of lung cancer. Chr: chromosome; DNAm: DNA methylation; HUNT: The Trøndelag Health Study.

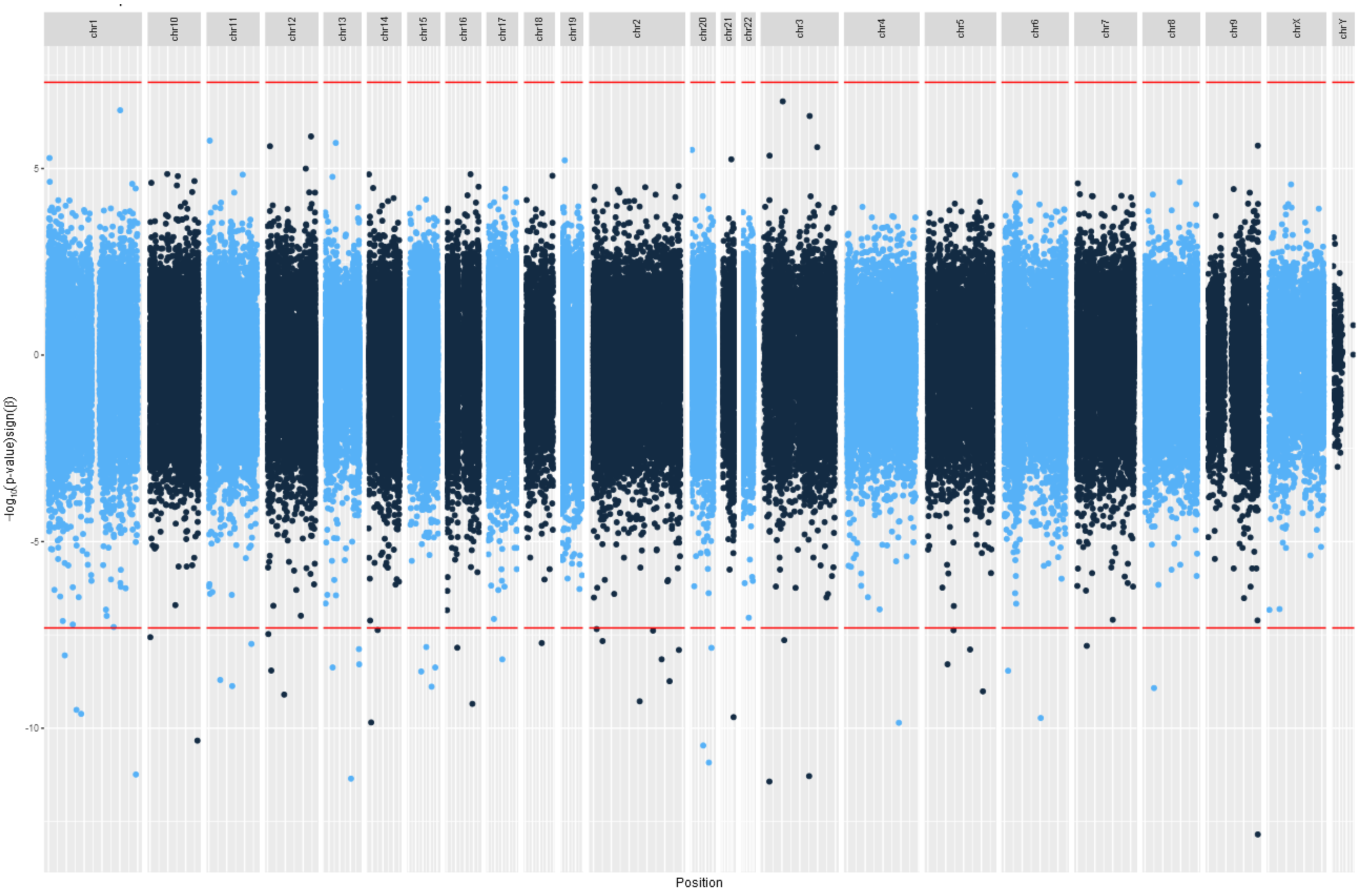

Supplementary Figure S6. Correlation between MR and EWAS estimates for associations between DNA methylation and risk of lung cancer for the 13 putative mediators. EWAS: epigenome-wide association study; MR: Mendelian randomization.

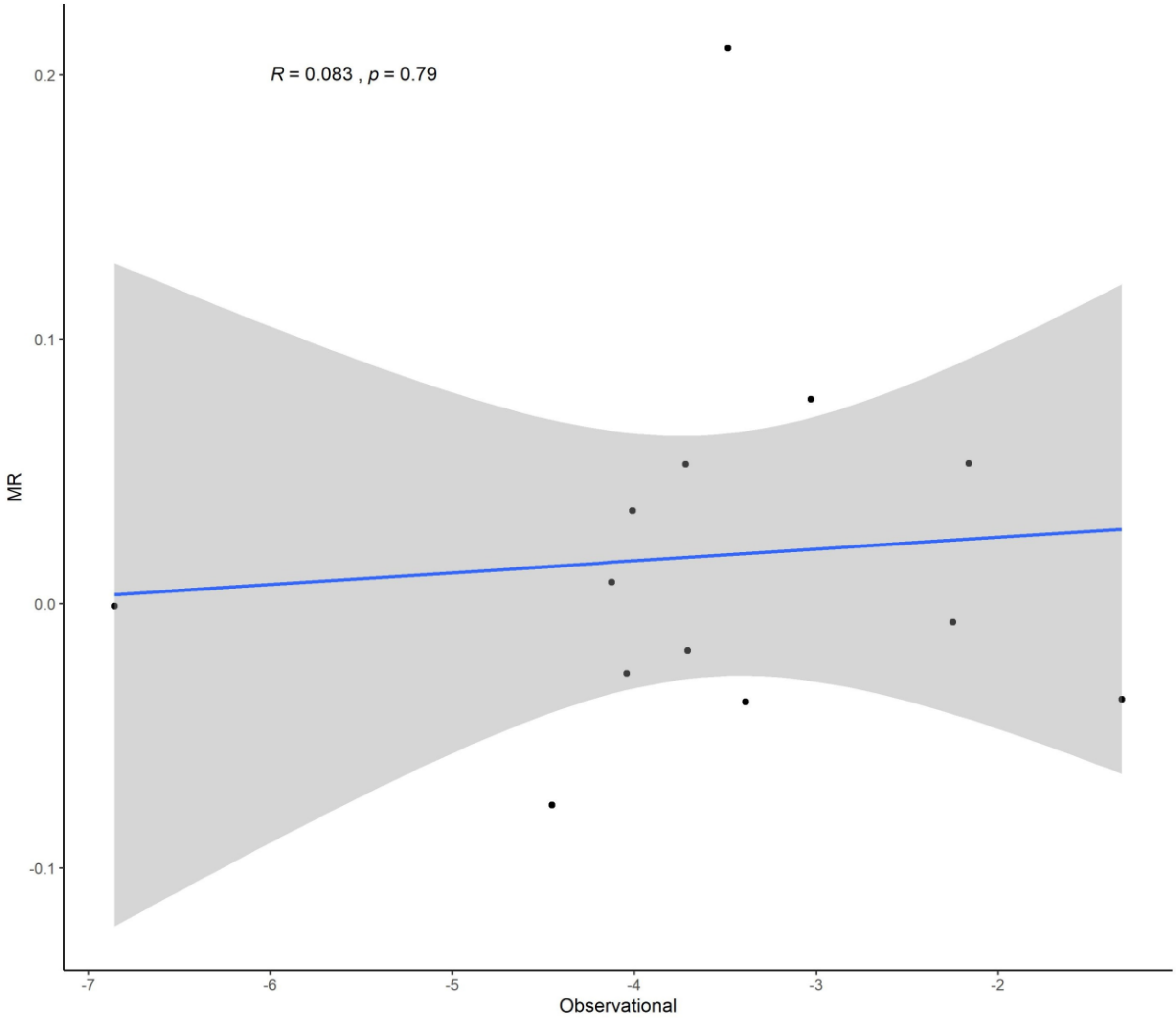

Supplement: dyab044_Supplementary_Data [file dyab044_supplementary_data.pdf]
